# New and Investigational Treatment Options for Dermatomycosis in the Era of Antifungal Resistance

**DOI:** 10.3390/jof12030221

**Published:** 2026-03-19

**Authors:** Aditya K. Gupta, Amanda Liddy, Tong Wang

**Affiliations:** 1 Division of Dermatology, Department of Medicine, Temerty Faculty of Medicine, University of Toronto, Toronto, ON M5S 1A1, Canada; 2 Mediprobe Research Inc., London, ON N5X 2P1, Canada; aliddy@mediproberesearch.com (A.L.); twang@mediproberesearch.com (T.W.)

**Keywords:** dermatomycoses, antifungal agents, drug development, investigational drug, vaccines, nanomedicine

## Abstract

Superficial mycoses (dermatomycoses) are a growing healthcare concern due to antifungal resistance, particularly among aging and immunocompromised populations. Multiple efforts are underway to develop novel antifungals, including discovering new compounds with known or new mechanisms of action, extending indications or repurposing existing medications, and utilizing vaccination and nanotechnology platforms. Herein, we conducted a scoping review of novel antifungals for the treatment of dermatomycoses. An electronic literature search restricted to the past 10 years was performed in January 2026 using PubMed and Embase (Ovid). Olorofim and ME1111 represent novel drug classes that target intracellular metabolism. New agents belonging to the azole class demonstrate reduced drug–drug interactions (oteseconazole), a broader antifungal spectrum (voriconazole), and reduced pharmacokinetic complexity (fosravuconazole, super-bioavailable itraconazole). Other investigational compounds include allicin, a phytocompound, and miltefosine, a repurposed antileishmanial drug. Based on our current understanding of dermatophyte immunity, antimicrobial peptides and vaccines targeting virulence factors (e.g., subtilisins) represent novel strategies. Nanotechnology platforms also show promise in introducing new antifungal agents (e.g., metal nanoparticles, nitric oxide-releasing nanoparticles), as well as developing topical formulations to enhance the bioavailability and safety profiles of existing antifungals (amphotericin B, ketoconazole, voriconazole).

## 1. Introduction

Dermatomycoses represent an increasing global healthcare burden, due in part to the emergence of antifungal resistance [1,2]. Initially reported as sporadic cases prior to 2015, the issue of terbinafine resistance has since escalated into a public health concern following the outbreak of *Trichophyton* (*T.*) *indotineae* infections, which have demonstrated intercontinental spread [3]. Although typically regarded as mild infections, the epidemiological landscape of dermatomycoses is evolving, as reflected by recent reports of widespread, recalcitrant infections and invasive, systemic infections, particularly among immunocompromised populations [4,5,6]. Moreover, pathogens previously considered uncommon in the Global North may be emerging in new geographical regions, possibly related to climate change [7]. These developments underscore the need to investigate alternative treatment strategies.

**Figure 1:**
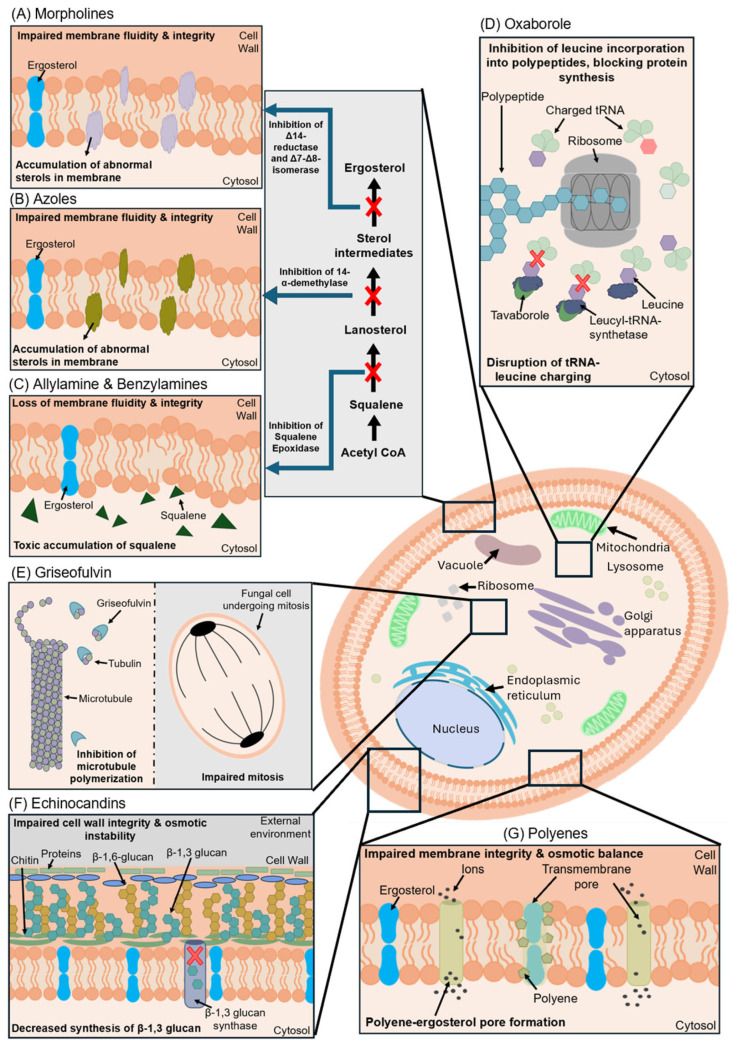
Mechanism of action of U.S. FDA-approved and novel antifungal agents. (**A**) Morpholines (amorolfine) inhibit Δ14-reductase and Δ7–Δ8-isomerase, which are involved in ergosterol biosynthesis, reducing ergosterol and leading to accumulation of abnormal sterols, which impair fungal cell membrane stability and integrity. (**B**) Azoles (itraconazole, fluconazole) inhibit 14-α-demethylase, decreasing ergosterol formation and resulting in buildup of demethylated sterol intermediates, leading to defective fungal cell membrane structure. (**C**) Allylamines (terbinafine) inhibit squalene epoxidase, resulting in lowered ergosterol levels and toxic squalene accumulation, which disrupts membrane integrity and permeability. (**D**) Oxaborole (tavaborole) inhibits fungal leucyl-tRNA synthetase, leading to accumulation of uncharged tRNA^Leu^ and impaired leucine incorporation into proteins, thereby blocking protein synthesis and resulting in growth arrest and cell death. (**E**) Griseofulvin binds fungal microtubules, inhibiting polymerization and mitotic spindle assembly, thereby blocking mitosis and fungal growth. (**F**) Echinocandins (caspofungin, micafungin) inhibit β-(1,3)-D-glucan synthase, preventing β-(1,3)-D-glucan synthesis and leading to fungal cell wall instability, osmotic stress, and lysis. (**G**) Polyenes (amphotericin B, nystatin) bind directly to ergosterol in the fungal cell membrane, forming pores that cause ion and metabolite leakage, resulting in osmotic imbalance and cell death. Figure was created using PowerPoint (version 1808).

A summary of antifungals currently approved by the U.S. Food and Drug Administration (FDA) is shown in Figure 1. The development of novel antifungals is a complex process that includes considerations of the drug target, pharmacodynamics (mode of action), pharmacokinetics including absorption, distribution, metabolism, and elimination, as well as dosage forms, off-target systemic toxicity concerns, and drug–drug interactions. For dermatomycosis, demonstration of in vitro efficacy (minimum inhibitory concentration [MIC]) alone does not accurately reflect clinical utility; rather, distribution, permeation, and retention in the stratum corneum relative to the MIC are also key indicators of efficacy [8,9]. Furthermore, agents with minimal risk of cross-resistance to terbinafine and azoles—such as those belonging to a new chemical class, with a novel target, and/or a new mode of action as per the World Health Organization's innovation criteria—are also relevant given the current global scenario [10].

Traditional approaches to developing antifungals can be broadly summarized as novel agents belonging to existing drug classes, agents with novel mechanisms of action, or formulation developments that address safety and/or bioavailability concerns. Off-label extension and drug repurposing are alternative strategies that reduce developmental costs, given that efficacy and safety profiles are already established, particularly for older agents with lapsed patents and without regulatory data protection. Recent technological advances may enable vaccination strategies as more virulence factors are discovered [11]. Nanoparticle systems can be applied either as new antifungal agents or as a topical delivery vehicle [12]. Beyond the scope of this review, non-pharmacological, device-based interventions such as photodynamic therapy theoretically bypass drug-resistance mechanisms and exert fungicidal effects via the localized induction of heat or oxidative stress [13]; furthermore, iontophoresis and ultrasound can enhance drug delivery across the nail plate for the treatment of onychomycosis [14]. In this scoping review, we collated literature from the past decade to provide a snapshot of novel antifungals under development for the treatment of dermatomycosis.

## 2. Materials and Methods

A scoping review was conducted on 21 January 2026 in accordance with the PRISMA recommendations (Open Science Framework: https://doi.org/10.17605/OSF.IO/JMC42 [accessed on 19 February 2026]) [15]. PubMed and Embase (Ovid) were searched with a restriction to publications from the past 10 years. The search strategy was based on the names or codes of investigational antifungals (from preclinical to clinical stages of development), as well as off-label antifungals not indicated for dermatomycoses, discussed in recently published reviews [16,17,18]. Emerging treatment modalities, including immunotherapies, vaccines, and nanomedicinal products, were also included. These terms were combined with the following keywords (including MeSH and Emtree terms): ‘dermatophytosis’, ‘dermatomycoses’, ‘fungal skin diseases’, ‘tinea’, or ‘ringworm’.

Search results were ported into Covidence (https://www.covidence.org/ [accessed on 21 January 2026]) for de-duplication and screening. The inclusion criteria were original studies of novel antifungals that demonstrated efficacy against dermatomycosis pathogens and/or showed favorable stratum corneum pharmacokinetics. Pharmacovigilance studies and reports on novel formulations relevant to dermatomycosis were also included. Novel agents supported by only in vitro susceptibility testing results were excluded. Non-pharmacological interventions (photodynamic therapy, laser) were also excluded. Fungal nomenclature was standardized according to recommendations by de Hoog et al. [19].

## 3. Results and Discussion

**Figure 2:**
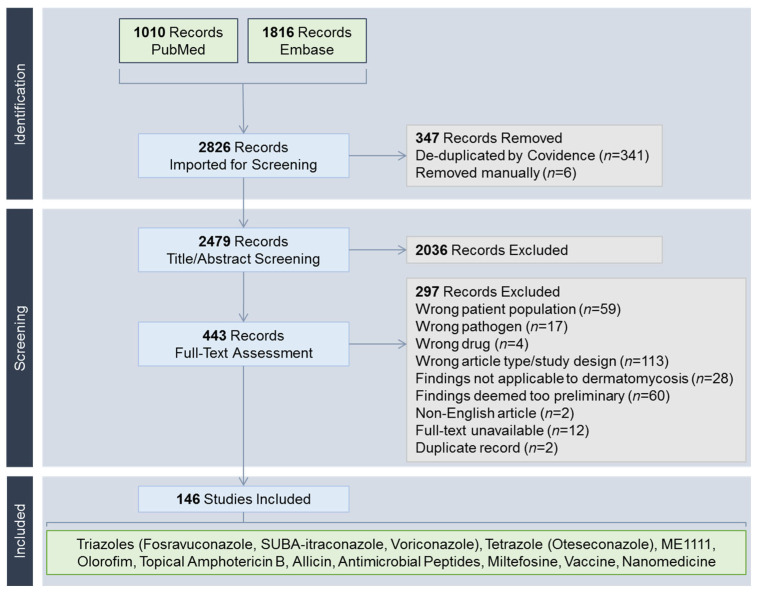
PRISMA flow diagram. SUBA-itraconazole, super-bioavailable itraconazole.

The search yielded 2826 records (Figure 2). Following full-text review, the following modalities were deemed to have sufficient pre-clinical and/or clinical evidence for discussion: fosravuconazole [20,21,22,23,24,25,26,27,28,29,30,31,32,33,34], super-bioavailable (SUBA) itraconazole [35,36,37,38,39,40,41,42,43,44,45], voriconazole [46,47,48,49,50,51,52,53,54,55,56,57,58,59,60,61,62,63,64,65,66,67,68,69,70,71,72,73,74,75,76,77,78,79,80,81,82,83,84,85,86,87,88,89,90,91,92,93,94,95,96,97,98,99,100,101,102,103,104,105,106,107,108,109,110,111,112,113,114,115,116], oteseconazole [117,118], ME1111 [119,120,121,122,123,124,125,126], olorofim [127,128,129], topical amphotericin B [130,131,132,133,134,135], allicin [136,137,138,139], antimicrobial peptides [140,141], miltefosine [142,143,144,145], vaccines [146], and nanomedicines [147,148,149,150,151,152,153,154,155,156,157,158,159,160,161,162,163,164,165]. A summary of newer antifungal compounds is shown in Table 1. Echinocandins (anidulafungin, caspofungin, micafungin, rezafungin) were not included due to their limited penetration into the stratum corneum relative to plasma concentrations, the necessity for parenteral administration, and the absence of novel formulation developments relevant to dermatomycosis [9].

### 3.1. Azoles

Azole antifungals exert their fungistatic effect through inhibition of sterol 14-α-demethylase (CYP51), a cytochrome P450 (CYP450) enzyme essential to ergosterol biosynthesis. By antagonizing this enzymatic step, azoles deplete the intracellular pool of ergosterol, thereby reducing the structural integrity and fluidity of the fungal cell membrane. Broadly, the clinical use of azoles faces issues of pharmacokinetic variability that can lead to supra- or subtherapeutic levels, as well as drug–drug interactions due to the off-target inhibition of human CYP450 enzymes, which can cause hepatotoxicity.

Triazoles—characterized by three nitrogen atoms in the azole ring (itraconazole, fluconazole)—represent the mainstay systemic treatment option for dermatomycoses [166]. By contrast, imidazoles—with two nitrogen atoms—generally show a greater risk of systemic toxicity and are therefore largely limited to topical use (e.g., ketoconazole, sertaconazole, fenticonazole, oxiconazole). Compared with triazoles, tetrazoles—with four nitrogen atoms—are designed to further reduce off-target effects on the human CYP51A isoform, thereby improving the safety profile. A summary of newer triazole and tetrazole agents is shown in Table 2.

**Table 1:** Summary of newer antifungal compounds for the treatment of dermatomycosis.

Compound (Drug Class)	Target	Clinical Trial ^a^	Phase (Population)	In Vitro Activity ^b^			Topical Formulations	Key Characteristics
				*Trichophyton*	*Microsporum*	*C. albicans*		
Fosravuconazole (Triazole)	ERG11/CYP51	Watanabe et al. [20] Okubo et al. [31] Naka et al. [32]	3 (onychomycosis) 4 (onychomycosis) 4 (onychomycosis)	✓	✓	✓	?	Reduced PK complexity
SUBA-ITZ (Triazole)	ERG11/CYP51	Dhoot et al. [36] Mohapatra et al. [41] Shah et al. [40] Shenoy et al. [43] Shenoy et al. [38,42] Tyagi et al. [45] Unpublished [167]	2 (glabrous tinea) 2 (glabrous tinea) 2 (glabrous tinea) 2 (glabrous tinea) 2 (glabrous tinea) 2 (glabrous tinea) 2 (onychomycosis)	✓	✓	✓	?	Reduced PK complexity
Voriconazole (Triazole)	ERG11/CYP51	Ali et al. [85] C.B.S. et al. [82] Khattab et al. [79] Shahzad et al. [83]	2 (glabrous tinea) 2 (glabrous tinea) 2 (glabrous tinea) 2 (glabrous tinea)	✓	✓	✓	[100,103,105,106,107,108,109,110,111,112,114]	Broad-spectrum antifungal activity
Oteseconazole (Tetrazole)	ERG11/CYP51	Elewski et al. [118]	2 (onychomycosis)	✓	?	✓	?	Higher specificity for fungal CYP51
ME1111	Mitochondria (Complex II)	?	-	✓	?	?	[120,122,123,126]	Novel drug target
Olorofim (Orotomide)	Pyrimidine Biosynthesis (DHODH)	?	-	✓	✓	No activity	[127]	Novel drug target
Allicin	Fungal cell and organelle membranes	?	-	✓	✓	✓	[139]	Phytocompound
NP213 (Antimicrobial peptide)	Fungal cytoplasmic membrane	Mercer et al. [140] Mercer et al. [140]	1 (onychomycosis) 2 (onychomycosis)	✓	✓	✓	[140,141]	Part of the host innate immunity
Miltefosine	Membrane lipids Mitochondria (Complex IV)	?	-	✓	?	✓	[142,143,144,145]	Repurposed antileishmanial medication

DHODH, dihydroorotate dehydrogenase; SUBA-ITZ, super-bioavailable itraconazole. “✓”: in vitro activity against dermatomycosis pathogens was reported. “?”: no relevant information was identified in this review. ^a^ Only relevant clinical trials conducted in dermatomycosis patients are shown. ^b^ In vitro antifungal susceptibility was summarized only for common dermatophytic pathogens; non-*albicans Candida* and non-dermatophyte molds (*Aspergillus*, *Fusarium*) are not shown. Note that in vitro susceptibility testing results should be interpreted with caution due to the lack of clinical breakpoints.

**Table 2:** Summary of newer azole antifungals for the treatment of dermatomycosis.

Agent (Dosage Form)	Approved Indication (Country)	PK/PD Efficacy Index *	Effects of Food	Drug–Drug Interactions
**Triazole**				
Fosravuconazole [168] (Capsules) 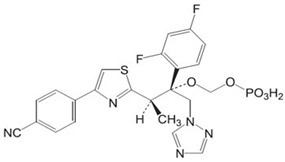	Onychomycosis caused by dermatophytes (Japan)	AUC/MIC (Linear)	No difference in systemic absorption (AUC_0−t_) between fed and fasted states	CYP3A substrates Warfarin
SUBA-ITZ ^†^ [167,169] (Capsules) 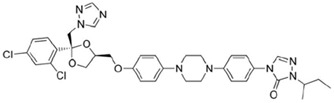	Invasive fungal infections: Blastomycosis (USA); Histoplasmosis (USA, Australia); Aspergillosis (USA, Australia); Candidiasis (Australia) Dermatomycoses and pityriasis (tinea) versicolor when external treatment is ineffective or inappropriate (Australia)	AUC/MIC (Non-linear)	High-fat meal decreases steady-state absorption (C_max_, AUC_tau_) *The steady-state pharmacokinetics of SUBA-ITZ 65 mg twice daily were similar to those of C-ITZ 100 mg twice daily*	CYP3A4 substrates Colchicine Fesoterodine Solifenacin Eliglustat
Voriconazole [170,171,172,173] (Tablets, Oral Suspension, IV injection) 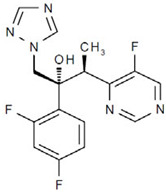	Serious fungal and yeast infections, such as aspergillosis, scedosporiosis, fusariosis, candidemia, and deep tissue or esophageal candidiasis ** Usage is primarily for patients with progressive, potentially life-threatening infections	AUC/MIC (Non-linear)	High-fat meal reduces absorption (C_max_, AUC_Τ_) *Voriconazole should be administered at least one hour before, or one to two hours after, a meal*	CYP2C19, CYP2C9, and CYP3A4 inducers or inhibitors CYP2C19, CYP2C9, and CYPA4 substrates
**Tetrazole**				
Oteseconazole [174] (Capsules) 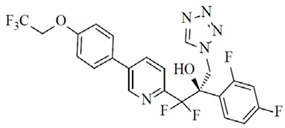	Recurrent vulvovaginal candidiasis in patients without reproductive potential (USA)	AUC/MIC (Linear)	High-fat meal increases absorption (C_max_, AUC_0–72h_)	BCRP substrates

AUC, area under the curve; BCRP, breast cancer resistance protein; C-ITZ, conventional itraconazole; C_max_, peak serum concentration; CYP, cytochrome p450; IV, intravenous; MIC, minimum inhibitory concentration; PD, pharmacodynamic; PK, pharmacokinetic; SUBA-ITZ, super bioavailable itraconazole. * The PK/PD index associated with efficacy is generally assumed to be AUC/MIC for triazoles and tetrazoles [17,175]. ** Invasive aspergillosis (USA, Canada, EU, UK); Candidemia in nonneutropenics (USA, Canada, EU, UK); Disseminated candidiasis in the skin, abdomen, bladder wall, and wounds (USA, Canada); Esophageal candidiasis (USA); Fluconazole-resistant invasive *Candida* infections including *C. krusei* (EU, UK); Scedosporiosis and fusariosis (USA, EU, UK); Prophylaxis of invasive fungal infections in high-risk hematopoietic stem cell transplant recipients (EU, UK). ^†^ In the USA, SUBA-itraconazole is not recommended for onychomycosis and should not be used as a substitute for other itraconazole formulations.

#### 3.1.1. Fosravuconazole

Fosravuconazole is a triazole and a prodrug of ravuconazole with improved lipophilicity and absorption while exhibiting reduced off-target effects on human CYP3A4 compared with itraconazole. Unlike itraconazole, its total systemic absorption (AUC_0−t_) is not affected by food intake [168]. Following oral administration, fosravuconazole is converted to ravuconazole, which accumulates in nails, reaching a concentration of 120.1 ng/g in the toenails of onychomycosis patients after receiving 100 mg/d for 12 weeks [168]. Beyond its use in onychomycosis, recent research has highlighted its utility in treating glabrous tineas, tinea barbae, tinea capitis, kerion celsi, and resistant cases [23,25,26,29,30,33,34].

In 2018, a fosravuconazole 100 mg capsule was approved for the treatment of onychomycosis in Japan [168]. In a phase 3 trial, 153 patients with moderate-to-severe onychomycosis—positive for *T. rubrum* or *T. mentagrophytes*—were randomized to receive either fosravuconazole 100 mg/d or placebo for 12 weeks [20]. At week 48, 59.4% (60/101) of patients receiving fosravuconazole achieved complete cure (clinical resolution with negative KOH microscopy) compared with 5.8% (3/52) in the placebo arm. Common drug-related adverse events were gastrointestinal symptoms [20]. Liver enzyme elevations (ALT, AST, γ-GTP) occurred at a rate of 18.8% (19/101), which resolved after treatment completion [20]. Follow-up studies have reaffirmed its efficacy in treating total dystrophic onychomycosis, as well as in hemodialysis patients, patients nonresponsive to topical antifungals, and infections complicated by dermatophytomas [21,24,27,28].

In onychomycosis patients with suboptimal response or experienced recurrence, retreatment with fosravuconazole 100 mg/d for 12 weeks was not associated with serious adverse events or new safety signals [31,32]. However, a retrospective analysis of 36 onychomycosis patients identified a risk of renal injury (27.8% [10/36]), defined as either an increase in serum creatinine by ≥0.3 mg/dL or ≥50%, or a decrease in estimated glomerular infiltration rate of ≥50% [22]. The exact cause is unclear; however, being on angiotensin-converting enzyme inhibitors or angiotensin II receptor blockers was identified as a risk factor [22].

#### 3.1.2. Super-Bioavailable (SUBA) Itraconazole

The SUBA technology was developed to incorporate itraconazole into a pH-dependent polymeric matrix, which enhances dissolution and targets drug release in the proximal small intestine. Since its absorption is not dependent on an acidic gastric environment, unlike conventional itraconazole, inter-patient pharmacokinetic variability is also reduced. Compared to conventional itraconazole, SUBA-itraconazole has a relative bioavailability of 173% with 21.3% less interpatient variability [176]. Furthermore, it exhibits higher tissue distribution to the stratum corneum compared to the deeper epidermis or dermis [37]. In the USA, a SUBA-itraconazole 65 mg capsule is approved for the treatment of invasive fungal infections, but it is not recommended for use in dermatomycosis [169]. By contrast, a SUBA-itraconazole 50 mg capsule is approved in Australia for the treatment of superficial and systemic mycoses [167]. In India, SUBA-itraconazole is actively investigated as a treatment for recalcitrant dermatophytosis, and is available as 50 mg, 65 mg, 100 mg, or 130 mg capsules [35,39].

In dermatomycosis patients administered either SUBA-itraconazole 130 mg/d or conventional itraconazole 200 mg/d for 4 weeks, similar serum and sebum concentrations were observed, suggesting bioequivalence [36]. Head-to-head studies confirmed a similar degree of clinical and mycological response in patients who received SUBA-itraconazole 100 mg/d or 130 mg/d compared to conventional itraconazole 200 mg/d, while a less favorable response was observed in patients who received conventional itraconazole 100 mg/d [38,43,45]. A randomized trial compared moderate-to-severe toenail onychomycosis patients receiving either SUBA-itraconazole 100 mg/d or conventional itraconazole 200 mg/d for 12 weeks [167]. At week 24, SUBA-itraconazole was shown to be non-inferior to conventional itraconazole. Another study demonstrated that SUBA-itraconazole 130 mg/d may be superior to SUBA-itraconazole 100 mg/d [40]; however, there are conflicting findings and opinions on the optimal starting dosage due to its non-linear pharmacokinetics [35,39,41]. Overweight or obesity (BMI ≥ 25 kg/m^2^) was not a significant factor in treatment response [42].

None of the identified studies in dermatomycosis patients reported serious adverse events or acute liver injuries. In a case report, an adolescent tinea cruris patient developed a probable case of drug eruption (intertriginous and flexural exanthema) after receiving SUBA-itraconazole 130 mg/d [44]. Regardless of formulation, itraconazole use also carries a risk of cardiac dysfunction due to possible mitochondrial toxicity [169]; in a retrospective case review, patients experiencing cardiac events—including edema, heart failure, and worsening or new hypertension—were mostly older adults (average age: 66 years) with an average serum drug level of 5.2 µg/mL [177]. Similar to conventional itraconazole, therapeutic drug monitoring (TDM) can help tailor dosage to prevent supra- or subtherapeutic levels and reduce systemic toxicity risks. Although SUBA-itraconazole may be taken with or without food [167], its bioavailability is reduced by 26.9% in the fed state compared to the fasted state, which can lead to variable clinical outcomes when food intake is not specified in treatment protocol [176]. The U.S. FDA still recommends that SUBA-itraconazole be taken with food [169].

#### 3.1.3. Voriconazole

Voriconazole is a broad-spectrum triazole agent with significant inter- and intrapatient pharmacokinetic variability (e.g., CYP2C19 polymorphisms, food intake, drug–drug interactions), which can lead to sub- or supratherapeutic drug levels and unpredictable treatment outcomes, warranting TDM and consultation with an infectious disease specialist. In 2002, voriconazole (tablets, oral suspension, intravenous injection) was approved in the USA for the treatment of invasive fungal infections, including those caused by *Aspergillus*, *Fusarium solani*, *Scedosporium apiospermum*, and *Candida* [170]. Although voriconazole tablets have recently been tried off-label for recalcitrant dermatomycosis, its utility is still debated due to high medication costs, lack of information on skin/nail pharmacokinetics, and safety concerns, including photosensitivity and photocarcinogenesis. To minimize systemic toxicity risks, recent research has developed topical voriconazole formulations with demonstrated permeation across the skin and nails.

Antifungal susceptibility testing has demonstrated broad-spectrum activity against isolates from dermatomycosis patients. For onychomycosis caused by non-dermatophyte molds, voriconazole has demonstrated efficacy against *Aspergillus* spp. and *Fusarium* spp. (*F. solani* species complex, *F. fujikuroi* species complex, *F. oxysporum*), including in strains with high terbinafine, griseofulvin, or fluconazole MICs [46,47,55,69]. *Candida* isolates from onychomycosis patients also exhibited high susceptibility, including *C. albicans* and non-*albicans Candida* (*C. parapsilosis*, *C. krusei*, *C. tropicalis*) [64,65,113]. Additionally, voriconazole was effective in vitro against dermatophytes [48,49,50,54,56,57,58,59,60,62,65,73,74,76,80,91,102,115,116], including *T. rubrum*, the *T. mentagrophytes* complex, and, importantly, *T. indotineae* resistant to standard antifungals [51,52,53,61,63,66,67,68]. However, a cautious approach is warranted, given that itraconazole resistance may be emerging in dermatophytes, which can confer cross-resistance to voriconazole [52,70,75,78,86,88,93]. The indiscriminate dual use of azoles in agriculture and medicine has been linked to resistance development in *Fusarium* [72]. A recent study also reported reduced voriconazole and fluconazole susceptibility in non-*albicans Candida* causing onychomycosis [71].

After serial dosing in a guinea pig model, voriconazole was detected in skin biopsies at concentrations nearly twice as high as in blood, while skin microdialysates exhibited lower concentrations than in blood [178]. Clinically, oral voriconazole has been tried successfully in recalcitrant glabrous tinea and onychomycosis, albeit with significant variations in dosage and duration, as well as unclear safety concerns such as abnormal vision and liver enzyme elevations requiring regular monitoring. In a randomized study, recalcitrant tinea corporis/faciei patients received a loading dose of voriconazole 800 mg on day 1, followed by 400 mg/d for a total of 6 weeks [79]. Compared with patients who received itraconazole 200 mg/d for 6 weeks, the voriconazole group achieved significantly higher mycological (negative culture and KOH microscopy; 86.7% [26/30] vs. 56.7% [17/30]) and complete cure rates (clinical resolution with mycological cure; 83.3% [25/30] vs. 53.3% [16/30]) by month 6, with abnormal vision occurring in 6.7% (2/30) of patients and without laboratory abnormalities [79]. A similar study of 40 patients reported clinical resolution after administering the same regimen for a shorter duration of 2 weeks [82]. In the absence of a loading dose, 200 mg twice daily and 200 mg/d were also tried successfully when administered for 2 weeks or more [81,83,85,93]. For onychomycosis patients, 200 mg twice daily for 3 months was tried for fingernails [77], and 200 mg twice daily on day 1 followed by 200 mg/d for 3–4 months was given to treat toenails [87]. Voriconazole was generally reported as well-tolerated with self-limiting adverse events; however, a large study of 227 tinea corporis/cruris patients receiving 200 mg twice daily for 2 weeks reported a 4.9% frequency of developing jaundice [99], reinforcing the issue of pharmacokinetic variabilities and the need for TDM to tailor dosage.

##### Safety Considerations and Novel Topical Formulations

TDM is strongly recommended for voriconazole due to its complex, non-linear pharmacokinetics [170]. Following oral administration, the maximum plasma concentration (C_max_) is reached after 1–2 h, with 96% bioavailability and extensive tissue distribution [170]. It is metabolized by CYP450 enzymes, particularly by CYP2C19, with voriconazole-*N*-oxide being the main metabolite that does not show significant antifungal activity [170]. Due to saturation of its metabolism, the rate of systemic exposure increases disproportionately with dose [170]. Furthermore, a significant portion of the population is poor metabolizers—15–20% in Asians, 3–5% in Caucasians and Blacks—attributed to CYP2C19 genetic polymorphisms [170]. The absorption of voriconazole is further complicated by food intake, and it is recommended to fast for at least one hour before and one to two hours after administration [170]. For treating invasive fungal infections, a serum concentration of ≥1.0 µg/mL is correlated with efficacy, while a supratherapeutic concentration of >4.0 µg/mL is correlated with hepatotoxicity [89,92].

In view of systemic toxicity concerns, topical voriconazole formulations offer a promising alternative. Due to its poor water solubility, the addition of penetration enhancers or a hydrophilic vehicle has been investigated [101]. Given the lipophilic nature of the stratum corneum, voriconazole loaded in a lipid matrix has been demonstrated to penetrate the stratum corneum down to the deep dermis [100]. A hydrogel formulation containing menthol (a lipophilic enhancer) has been shown to improve both the solubility and skin permeability of voriconazole, with in vitro efficacy against fluconazole-resistant *Candida albicans* [103]. A voriconazole 1% cream was also tried successfully in a terbinafine-resistant *T. indotineae* patient with an intracutaneous drug concentration of 0.5 µg/mL [114].

To maximize drug-tissue contact and improve drug-release kinetics, nanoparticle-based delivery systems have been explored for topical voriconazole administration. This includes chitosan-based nanoparticles [105], ethosomes [106,107], hyalurosomes [108], invasosomes [109], and transethosomes [110]. A liposomal formulation has demonstrated in vitro efficacy against *T. rubrum*, with skin permeation and follicular retention [111]. For nail delivery, the addition of thioglycolic acid—a reducing agent targeting the disulfide bonds in hard keratins—into a lipid-based formulation (nanomicelles) significantly increased drug retention by softening and hydrating the nail plate and disrupting the lipid barrier [112].

Photosensitivity reactions are another safety concern for voriconazole, with an almost 50-fold higher odds compared to other medications according to the U.S. FDA Adverse Events Reporting System [90]. Through its chromosomal localization that disrupts the initiation of DNA nucleotide excision repair, prolonged voriconazole use is linked to benign and malignant skin neoplasms, including nevi, actinic keratosis, and squamous cell carcinoma (SCC) [94]. The risk of SCC is further increased in rapid CYP2C19 metabolizers; this is postulated to result from the photoreaction of voriconazole-*N*-oxide that induces oxidative DNA damage [95]. The risk quantification remains unclear for the treatment of dermatomycosis, as voriconazole-related SCC cases generally occur in immunocompromised populations following prolonged treatment [96,97,98]. Consequently, patients receiving voriconazole should be advised to minimize direct sunlight exposure and use photoprotection [170].

#### 3.1.4. Oteseconazole

Oteseconazole (VT-1161) is a tetrazole agent that demonstrated greater target specificity for fungal CYP51. Both itraconazole and oteseconazole showed similar binding affinities for CYP51 from *T. rubrum*—preventing the demethylation of eburicol—compared with fluconazole and ketoconazole [117]. Similar findings were shown with *Candida albicans* CYP51 [179]. Furthermore, oteseconazole showed no evidence of binding to the heme iron of human CYP51, in contrast to clotrimazole, fluconazole, itraconazole and voriconazole [179].

In the USA, oteseconazole was approved in 2022 for the treatment of recurrent vulvovaginal candidiasis (RVVC) in patients without reproductive potential [174]. Across three phase 3 trials, RVVC patients receiving oteseconazole—either alone or sequentially with fluconazole—were significantly less likely to experience acute episodes than those receiving placebo [174]. In a phase 2 trial evaluating oteseconazole for the treatment of distal lateral subungual onychomycosis [118], a total of 259 patients were randomized to receive a 2-week induction dose of either 300 mg/d or 600 mg/d, followed by a weekly maintenance dose of either 300 mg or 600 mg, respectively, for 10 or 22 weeks. Compared to the placebo arm, patients receiving oteseconazole achieved significantly higher complete cure rates (clinical resolution with a negative KOH microscopy and culture; 40.7–45.3%) at week 60 [118]. Treatment-related adverse events were primarily gastrointestinal symptoms, with no abnormalities in liver function parameters or QT intervals [118].

### 3.2. ME1111

**Figure 3:**
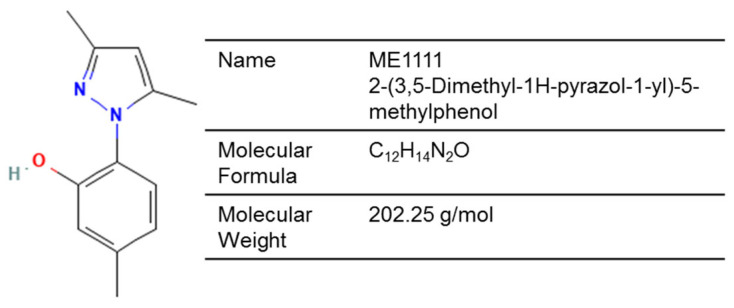
Chemical structure of ME1111 (PubChem Identifier: 60143882).

ME1111 is being investigated as a topical onychomycosis treatment due to its favorable nail pharmacokinetics and antifungal effects—via inhibition of succinate dehydrogenase (complex II)—that disrupt mitochondrial function (Figure 3) [119,121]. Its 10% solution has shown improved permeation of full-thickness human nails ex vivo when compared with ciclopirox 8% or amorolfine 5% [120]. After repeated once-daily applications for 14 days, significant distribution was detected in the ventral nail plate and the subungual space of human toenails and fingernails [122,123]. Distribution to the stratum corneum was also demonstrated in a guinea pig model [126].

Antifungal susceptibility testing has demonstrated its in vitro efficacy against *T. rubrum*, *T. mentagrophytes* complex, *T. tonsurans*, and *Epidermophyton floccosum* [120,124]. In a guinea pig model infected with the *T. mentagrophytes* complex, ME1111 10% applied once daily for 7 days induced significant clinical and mycological response compared with placebo [125], and exhibited a dose-dependent relationship [126].

### 3.3. Olorofim (F901318)

**Figure 4:**
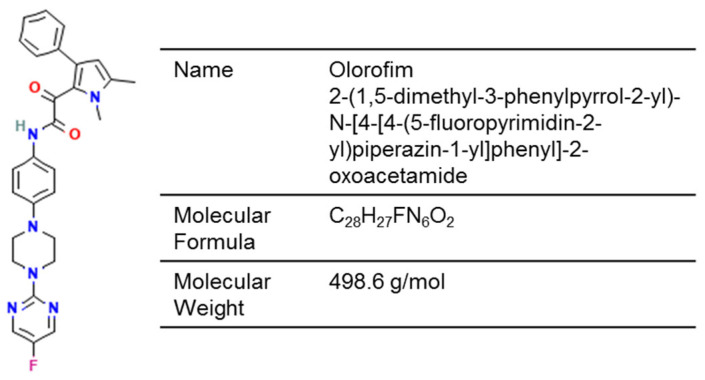
Chemical structure of olorofim (PubChem Identifier: 91885568).

Olorofim is an investigational orotomide compound with activity against a specific spectrum of molds and dimorphic fungi, but not yeasts and *Mucorales* (Figure 4) [175]. It disrupts pyrimidine biosynthesis by inhibiting fungal dihydroorotate dehydrogenase (DHODH), leading to downstream fungicidal effects including inhibition of germination and hyphal growth, cell wall disruption, and cell cycle arrest.

Initially developed as a treatment for azole-resistant *Aspergillus fumigatus* [180], olorofim may also bind to dermatophyte DHODH due to overlap in the conserved amino acid residues required for binding [127]. In a guinea pig model, olorofim was effective in treating dermatophytosis caused by *Nannizia gypsea* [127]. Olorofim also demonstrated favorable in vitro efficacy against *Trichophyton* spp. and *Microsporum canis*, including strains exhibiting terbinafine and itraconazole resistance, as well as *T. indotineae* [128,129].

In the treatment of invasive fungal infections, the antifungal activity of olorofim is time-dependent, as reflected by its C_min_/MIC [175]. Olorofim may cause drug–drug interactions due to its metabolic pathway involving multiple CYP450 enzymes and its inhibition of CYP3A4 [175]. In a phase 1 trial of eight healthy volunteers, olorofim tablets (360 mg/d for 10 days) did not lead to serious adverse events and the average plasma trough level was 1–2 µg/mL, albeit with liver enzyme elevation (ALT) occurring in one patient [181].

### 3.4. Topical Amphotericin B

Polyenes, such as amphotericin B, exhibit a broad spectrum of fungicidal activity by binding to ergosterol, which forms transmembrane pores and leads to the leakage of intracellular contents. However, the clinical use of amphotericin B is significantly limited by systemic toxicity, particularly nephrotoxicity. Although lipid-based formulations have improved its safety profile, issues related to stability, bioavailability, and the need for parenteral administration—as well as limited tissue distribution in the skin—restricts their use in dermatomycosis patients [9].

Topical delivery strategies offer a promising alternative; however, their development is complicated by the compound’s high molecular weight, which impedes its penetration across the skin barrier. A deformable liposomal amphotericin B formulation was developed, incorporating an edge activator to increase membrane elasticity, allowing the liposomes to pass through smaller pores [130]. Using human skin explants, this formulation exhibited permeation across the stratum corneum and into the deeper epidermis [130]. Nano-scaled lipid carriers for amphotericin B have also demonstrated in vitro efficacy against *Trichophyton* spp., including *T. indotineae* [131,132]. To further facilitate skin permeation, a dissolvable microneedle patch was developed, allowing permeation across both the epidermal and dermal layers [135].

For treating onychomycosis, Souza et al. incorporated amphotericin B into a commercial nail lacquer, demonstrating enhanced nail permeation compared to a control solution (amphotericin B in dimethyl sulfoxide), as well as in vitro efficacy against *Candida* spp. (*albicans* and non-*albicans*) [133]. In a pilot study, 15 onychomycosis patients were treated with a topical nanoliposomal amphotericin B 0.4% gel, applied twice daily for 12 weeks for fingernails or 36 weeks for toenails [134]. At week 36, 73.3% (11/15) of patients achieved complete cure (clinical resolution with negative KOH microscopy and culture), with application-site adverse events including transient nail discoloration occurring in all treated patients and onycholysis occurring in one patient [134].

### 3.5. Other Investigational Agents

#### 3.5.1. Allicin

**Figure 5:**
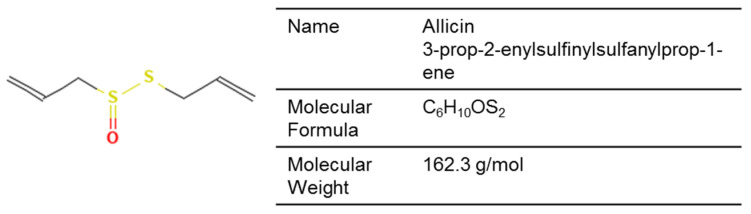
Chemical structure of allicin (PubChem Identifier: 65036).

Allicin is a sulfur-based phytocompound derived from garlic that has demonstrated fungistatic effects in vitro against dermatophytes and *Candida*, including disruption of germination and hyphal growth in *Trichophyton* spp., as well as protective effects on keratinocytes (Figure 5) [182]. In *Candida guilliermondii* and *Rhodotorula mucilaginosa* isolates from an onychomycosis patient, allicin penetrated cell and organelle membranes, resulting in cytoplasmic disruption and nuclear destruction [138]. Additionally, in keratinocytes challenged by reactive oxygen species (ROS), allicin demonstrated a protective effect against apoptosis and attenuated the pro-inflammatory response [137].

In *Microsporum canis* isolates from tinea capitis patients, allicin exhibited synergistic interactions with terbinafine and itraconazole [136]. A new formulation delivering allicin in a nanoparticle carrier mixed with hydrogels has been described, which may improve skin permeability and release kinetics for topical applications [139].

#### 3.5.2. Antimicrobial Peptides

**Figure 6:**
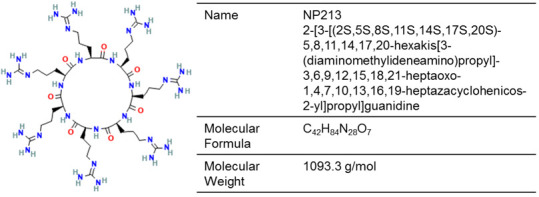
Chemical structure of NP213 (PubChem Identifier: 16679727).

Keratinocytes secrete antimicrobial peptides as part of the inmate immune response against dermatophytes [183]. Based on antimicrobial peptides found in onychomycosis patients, NP213 was developed for topical application, demonstrating effective nail permeation due to its cationic property and resistance to proteases, as well as in vitro fungicidal activity against *T. rubrum* through disrupting the cytoplasmic membrane (Figure 6) [141]. In a phase 2a trial, topical NP213 was administered once daily in 47 onychomycosis patients for 4 weeks [140]. Based on the per-protocol population, patients receiving NP213 achieved a significantly higher mycological cure rate (culture-negative) of 56.5% (13/23) by month 12 compared to no cured cases with the placebo.

#### 3.5.3. Miltefosine

**Figure 7:**
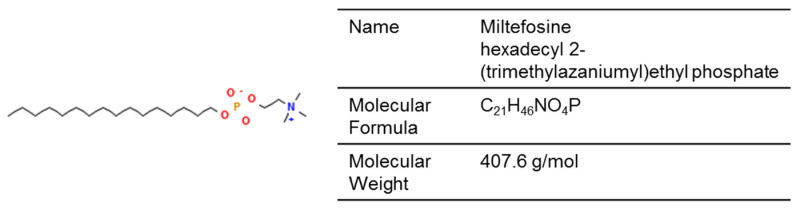
Chemical structure of miltefosine (PubChem Identifier: 3599).

Miltefosine, an alkyl phospholipid compound originally developed as a cancer treatment, was repurposed for the treatment of leishmaniasis in 2014 (Figure 7) [184]. Its proposed mechanisms of action include interactions with membrane lipids, ROS generation via the inhibition of cytochrome c oxidase, and induction of apoptosis [184]. Miltefosine has also demonstrated therapeutic potential against infections caused by *Fusarium*, *Candida*, and dermatophytes [185,186,187]. However, its oral administration is complicated by gastrointestinal side effects.

A nanoparticle-based formulation (miltefosine niosomal gel 1%)—with improved stability and drug-release kinetics—was developed and tested in a guinea pig model of *T. indotineae* infection [142]. Compared to untreated animals or those treated with the vehicle or oral terbinafine, miltefosine niosome gel 1% led to improved clinical and mycological responses, corroborated by histopathologic examination [142]. Other similar formulations under development include a dissolvable microneedle patch for intradermal administration of niosomes, as well as topical transfersome and lipid-based preparations [143,144,145].

### 3.6. Vaccines

Although fungal antigens have been discovered, development of antifungal vaccines faces challenges related to cost-effectiveness and uncertain protection in immunocompromised populations in whom invasive infections are more frequent [11]. For dermatophytes, the existence of natural immunity is supported by in vitro evidence of CD4^+^ and CD8^+^ T-cell proliferation and cytotoxic activity in patients recently recovered from acute dermatophytosis, which clinically manifests as a delayed-type hypersensitivity reaction [188]. Similarly, dermatophyte reinfection in animals has been associated with faster disease clearance and infiltration of peripheral blood mononuclear cells [189].

Given the increasing global prevalence of dermatomycosis—estimated to have affected 1.7 billion individuals in 2021—vaccine development for the general population could represent a cost-effective strategy [1]. Furthermore, therapeutic vaccines may help curb the spread of antifungal-resistant species, such as *T. indotineae* [4], by augmenting the host immune response to aid antifungal treatments. Using live and inactivated vaccines prepared from crude extracts of *T. mentagrophytes*, *T. verrucosum*, and *Microsporum canis*, Abo-Elyazeed et al. demonstrated a protective response in a guinea pig model, evidenced by the induction of dermatophyte-specific IgG and delayed-type skin hypersensitivity reactions [146]. Further isolation of protein fractions containing exo-keratinases confirmed their antigenic role in eliciting immune responses in vaccinated animals [146].

Subtilisin 6, also known as Tri r 2, is a serine protease expressed by *Trichophyton* spp. during in vivo infection and has been shown to elicit a protective T-cell response, as evidenced by delayed-type hypersensitivity reactions [190,191,192]. Notably, an immunodominant epitope was identified that induced lymphoproliferation and interferon-gamma (IFN-γ) secretion from peripheral blood mononuclear cells in healthy individuals, suggesting its potential as a vaccine target [193]. Further genomic investigations may help in developing a polyvalent vaccine that broadly targets the subtilisin family of endopeptidases [194].

### 3.7. Nanomedicines

In dermatomycosis, the site of infection—stratum corneum—presents a challenge for both drug penetration and retention. While topical antifungals are commonly used, their utilities may vary when applied on densely packed, hard keratins like nails or hyperkeratotic skin, and they are generally used in patients without extensive skin involvement. In contrast, oral antifungals like terbinafine and itraconazole distribute through the sebum and are retained in the skin by adhering to keratins, while fluconazole penetrates the stratum corneum via diffusion from the capillaries [8]. However, oral antifungals carry systemic toxicity risks and drug–drug interactions. Nanotechnology—involving the manipulation of particles ranging from 1 to 100 nm in size—is an active area of research that can be applied either directly, as new antifungals (e.g., metal nanoparticles), or in the formulation of traditional antifungals to improve their pharmacokinetics (e.g., liposomes) [12].

Metal nanoparticles have been investigated for their direct fungicidal mechanisms, potentially through the induction of oxidative stress. This strategy shows promise in bypassing common antifungal resistance mechanisms, such as alterations in ergosterol biosynthesis enzymes (e.g., squalene epoxidase mutations and terbinafine resistance); however, it remains uncertain whether they can be effective as monotherapies. Against *T. rubrum*, topically applied nanoparticles that release nitric oxide have demonstrated efficacy in a mouse model as well as in vitro synergistic interactions with efinaconazole [152,160]. Application of silver and zinc oxide nanoparticles has been shown to inhibit the in vitro growth of the *T. mentagrophytes* complex and *T. verrucosum*, likely by reducing keratinase activity, preventing ergosterol synthesis, and disrupting the integrity of the mycelial network [159]. For cutaneous candidiasis, a gold nanoparticle solution demonstrated a similar degree of clinical efficacy in infected mice compared with topical nystatin [165].

For glabrous tineas, nanoparticle systems for antifungals currently indicated for oral or intravenous routes of administration with safety concerns—such as voriconazole (see Section Safety Considerations and Novel Topical Formulations) and amphotericin B (see Section 3.4)—are being actively investigated. Furthermore, in a mouse model, a ketoconazole-based nanoparticle system was developed for intradermal administration [150], as the oral formulation has been restricted or discontinued due to idiosyncratic hepatotoxicity. Compared to the topical ketoconazole 2% cream, this formulation showed increased dermal drug retention without significant inflammatory infiltrations [150]. Similar efforts have been reported in developing ketoconazole in nanoparticle gel formulations, terbinafine and itraconazole in lipid nanoparticles, and itraconazole nanocrystals in a dissolvable microneedle patch [153,154,155,156,157].

For onychomycosis, nanoparticle systems for ciclopirox, efinaconazole, ketoconazole, and griseofulvin have demonstrated enhanced nail permeation and ex vivo efficacy against nails infected with *T. rubrum*, the *T. mentagrophytes* complex, and/or *Candida albicans* [147,148,151,162]. Lipid nanoparticles, chitosan nanoparticles, and hydrogel embedded with polymeric nanospheres have been designed to incorporate terbinafine [158,163,164]. In a randomized, double-blind trial, 20 onychomycosis patients applied terbinafine-loaded lipid nanoparticle 1% gel twice daily for 2 months [161]. Compared to patients who received the standard terbinafine 1% cream or vehicle, a significant increase in clinical improvement was observed, albeit without differences in mycological response, possibly due to terbinafine-resistant strains [161].

Interestingly, nanoparticles may also be applied as a disinfectant against dermatophytes. In an effort to prevent infection recurrence or relapse, sock pieces coated with a zinc oxide nanoparticle 2% solution were incubated with dermatophyte isolates from tinea pedis patients [149]. A reduced number of viable isolates was observed compared to the control without co-incubation of sock pieces [149].

With growing interest in this field, the toxicity profile of nanomedicinal products remains to be fully examined. In addition to possible hypersensitivity reactions, metal nanoparticles can induce oxidative cellular damage associated with neurotoxicity and nephrotoxicity [195,196]. Accidental exposure to aerosolized nanoparticles—such as through inhalation or dermal absorption (skin and eyes)—may be associated with chronic toxicity [197]. Further mechanistic and clinical evaluations are warranted.

## 4. Conclusions

Our review of the current literature shows multiple novel avenues for developing dermatomycosis treatments, which can be categorized as (a) agents with innovative modes of action targeting intracellular metabolism (olorofim, ME1111) or inducing oxidative stress ([topical] nanoparticles); (b) a tetrazole agent with reduced drug–drug interactions (oteseconazole); (c) new and off-label extensions of triazole agents with reduced pharmacokinetic complexities (fosravuconazole, SUBA-itraconazole) or a broader antifungal spectrum (voriconazole); (d) a repurposed antileishmanial drug (miltefosine); (e) an antimicrobial peptide derived from the natural host immune response (NP213); (f) a biologically active phytocompound (allicin); (g) a vaccination strategy targeting dermatophyte virulence factors (keratinases); and (h) topical formulation developments—some incorporating nanotechnology—that potentially enable the safer use of antifungals while increasing bioavailability (amphotericin B, ketoconazole, voriconazole).

A universal challenge in developing traditional antifungal compounds is the risk of toxicity due to similarities in the cellular and biochemical features of humans and fungi. To this end, novel agents targeting virulence factors (e.g., subtilisins), therapeutic vaccines, or other immunotherapies (e.g., IFN-γ) show promise. For treating resistant strains, agents that block resistance mechanisms—such as efflux pump inhibitors or heat shock protein 90 inhibitors—represent a novel adjuvant treatment strategy not covered in the present review [198,199]. In contrast to the antifungal development pipeline for invasive fungal infections, topical agents have a unique role in the treatment of dermatomycoses as they may enhance bioavailability while posing a lower risk of systemic toxicity and drug–drug interactions, warranting further research into nanoparticle systems.

Although the worldwide emergence of antifungal resistance calls for the expansion of our therapeutic arsenal, identifying microbiological resistance in dermatophytes is complicated due to the lack of clinical breakpoints, which hinders the evaluation of cross-resistance risks for novel antifungals. Recent epidemiological cut-off values proposed for the standard broth microdilution method, using protocols by the Clinical & Laboratory Standards Institute and the European Committee on Antimicrobial Susceptibility Testing, can help in the preclinical evaluation of drug candidates [52,200]. When novel agents enter clinical testing, variations in trial design—such as endpoint definitions (e.g., mycological cure determined by direct microscopic examination and/or culture) and follow-up intervals—can limit comparability between studies. The former reflects ongoing limitations in fungal testing capacity across healthcare settings, including, but not limited to, resource-constrained settings. Technological advances such as real-time PCR, which do not rely on obtaining a culture, may improve the efficiency of patient selection and outcome evaluation. Overall, more work is needed to establish a clear regulatory pathway as more novel antifungals enter the developmental pipeline. This review is also limited by potential publication bias, as trial results may remain unpublished and drug candidates may be terminated without notice. Greater transparency and collaboration should be encouraged to allow more drug candidates to enter the clinical development pipeline.

## Data Availability

No new data were created or analyzed in this study. Data sharing is not applicable to this article.

## References

[1] Li D., Fan S., Zhao H., Song J., Guo L., Li W., Xu X., Li Q. (2025). Worldwide Trends and Future Projections of Fungal Skin Disease Burden: A Comprehensive Analysis from the Global Burden of Diseases Study 2021. Front. Public Health.

[2] Lockhart S.R., Chowdhary A., Gold J.A.W. (2023). The Rapid Emergence of Antifungal-Resistant Human-Pathogenic Fungi. Nat. Rev. Microbiol..

[3] Rhodes J., Hui S.T., Dellière S., Summerbell R.C., Scott J.A., Kaur A., Barton R.C., Leitao R., Hemmings S., Goiriz R. (2026). Emerging Terbinafine-Resistant *Trichophyton indotineae* between 2018 and 2023: A Multinational Genomic Epidemiology Study. Lancet Microbe.

[4] Khurana A., Sharath S., Sardana K., Chowdhary A. (2024). Clinico-Mycological and Therapeutic Updates on Cutaneous Dermatophytic Infections in the Era of *Trichophyton indotineae*. J. Am. Acad. Dermatol..

[5] Galili E., Barzilai A., Lev A., Amit S., Barel O., Lubitz I., Gazit Z., Lyakhovitsky A., Somech R., Shemer A. (2025). Genetic, Immunological and Clinical Assessment of Isolated Chronic Recalcitrant Dermatophytosis: A Prospective Study. Br. J. Dermatol..

[6] Gupta A.K., Wang T., Susmita, Saunte D.M.L., Hay R., Piguet V. (2025). Deep and Disseminated Dermatophytosis in Immunocompromised Populations—A Systematic Review. J. Eur. Acad. Dermatol. Venereol..

[7] Gupta A.K., Thornbush M., Wang T. (2025). Climate Change, Natural Disasters, and Cutaneous Fungal Infections. Int. J. Dermatol..

[8] Sardana K., Arora P., Mahajan K. (2017). Intracutaneous Pharmacokinetics of Oral Antifungals and Their Relevance in Recalcitrant Cutaneous Dermatophytosis: Time to Revisit Basics. Indian J. Dermatol. Venereol. Leprol..

[9] Felton T., Troke P.F., Hope W.W. (2014). Tissue Penetration of Antifungal Agents. Clin. Microbiol. Rev..

[10] WHO Antimicrobial Resistance Division Antifungal Agents in Clinical and Preclinical Development. https://www.who.int/publications/b/77087.

[11] Casadevall A. (2025). Fungal Vaccines: So Needed, so Feasible, and yet so Far Off. J. Clin. Investig..

[12] Ren M.-Y., Shi Y.-J., Ding Y., Lu W., Fan S.-S., Tao X.-H. (2023). Current Status and Research Progress of Nanoparticle Application in Superficial Fungal Infection. Eur. Rev. Med. Pharmacol. Sci..

[13] Robres P., Aspiroz C., Rezusta A., Gilaberte Y. (2015). Usefulness of Photodynamic Therapy in the Management of Onychomycosis. Actas Dermosifiliogr..

[14] Gupta A.K., Polla Ravi S., Choi S.Y., Konda A., Cooper E.A. (2023). Strategies for the Enhancement of Nail Plate Permeation of Drugs to Treat Onychomycosis. J. Eur. Acad. Dermatol. Venereol..

[15] Tricco A.C., Lillie E., Zarin W., O’Brien K.K., Colquhoun H., Levac D., Moher D., Peters M.D.J., Horsley T., Weeks L. (2018). PRISMA Extension for Scoping Reviews (PRISMA-ScR): Checklist and Explanation. Ann. Intern. Med..

[16] Akinosoglou K., Papageorgiou D., Gogos C., Dimopoulos G. (2025). An Update on Newer Antifungals. Expert Rev. Anti. Infect. Ther..

[17] Branda F., Petrosillo N., Ceccarelli G., Giovanetti M., De Vito A., Madeddu G., Scarpa F., Ciccozzi M. (2025). Antifungal Agents in the 21st Century: Advances, Challenges, and Future Perspectives. Infect. Dis. Rep..

[18] Lu X., Zhou J., Ming Y., Wang Y., He R., Li Y., Feng L., Zeng B., Du Y., Wang C. (2025). Next-Generation Antifungal Drugs: Mechanisms, Efficacy, and Clinical Prospects. Acta Pharm. Sin. B.

[19] de Hoog S., Walsh T.J., Ahmed S.A., Alastruey-Izquierdo A., Alexander B.D., Arendrup M.C., Babady E., Bai F.-Y., Balada-Llasat J.-M., Borman A. (2023). A Conceptual Framework for Nomenclatural Stability and Validity of Medically Important Fungi: A Proposed Global Consensus Guideline for Fungal Name Changes Supported by ABP, ASM, CLSI, ECMM, ESCMID-EFISG, EUCAST-AFST, FDLC, IDSA, ISHAM, MMSA, and MSGERC. J. Clin. Microbiol..

[20] Watanabe S., Tsubouchi I., Okubo A. (2018). Efficacy and Safety of Fosravuconazole L-lysine Ethanolate, a Novel Oral Triazole Antifungal Agent, for the Treatment of Onychomycosis: A Multicenter, Double-blind, Randomized Phase III Study. J. Dermatol..

[21] Shimoyama H., Yo A., Sei Y., Kuwano Y. (2021). Treatment Outcome with Fosravuconazole for Onychomycosis. Mycopathologia.

[22] Shinzato T., Nagai K., Hoshino Y., Fujiwara Y., Yamamoto Y., Ogura K., Morishita A., Okawa T., Ito K., Murakami M. (2025). Kidney Injury Associated with Fosravuconazole L-Lysine Ethanolate. Clin. Exp. Nephrol..

[23] Ohara S., Noguchi H., Matsumoto T., Hayashi D., Kashiwada-Nakamura K., Kubo M., Kano R., Fukushima S. (2025). Topical Luliconazole Treatment for Hyperkeratotic Tinea Pedis. Mycopathologia.

[24] Uchida H., Kamata M., Ishikawa T., Ito M., Watanabe A., Egawa S., Hiura A., Fukaya S., Hayashi K., Fukuyasu A. (2023). Safety and Effectiveness of Fosravuconazole for the Treatment of Onychomycosis in Haemodialysis Patients: A Single-Centre Retrospective Study. J. Eur. Acad. Dermatol. Venereol..

[25] Takeshima R., Asahina Y., Yaguchii T., Sato T. (2020). Tinea Barbae Due to *Trichophyton rubrum* Successfully Treated Using Oral Fosravuconazole L-Lysine Ethanolate. J. Dermatol..

[26] Kitauchi Y., Kumagai Y., Inoue-Masuda Y., Sugiura M., Sato T., Yaguchi T., Yokoyama T. (2021). Tinea Corporis Caused by Terbinafine-Resistant *Trichophyton rubrum* Successfully Treated with Fosravuconazole. J. Dermatol..

[27] Inoue T., Watabe D., Tsunemi Y., Amano H. (2023). Outcome of Fosravuconazole Treatment for Onychomycosis Refractory to Topical Antifungal Agents. J. Dermatol..

[28] Noguchi H., Matsumoto T., Kimura U., Hiruma M., Kano R., Kubo M., Fukushima S., Ihn H. (2021). Fosravuconazole to Treat Severe Onychomycosis in the Elderly. J. Dermatol..

[29] Shimoyama H., Taira H., Satoh K., Tamura T., Yo A., Sei Y., Makimura K., Kuwano Y. (2023). Kerion Celsi Due to *Microsporum canis* in an Adult Woman, Treated Successfully with Fosravuconazole. Med. Mycol. J..

[30] Suzuki T., Sato T., Kasuya A., Yaguchi T. (2021). A Case of Tinea Faciei, Tinea Corporis, and Tinea Unguium with Dermatophytoma Successfully Treated with Oral Fosravuconazole L-Lysine Ethanolate. Med. Mycol. J..

[31] Okubo A., Hanada M., Kodama S., Taniguchi N., Miyazaki Y. (2024). Long-term Follow-up Study of the Efficacy of Fosravuconazole in the Treatment of Onychomycosis in Elderly Patients. J. Dermatol..

[32] Naka W., Tsunemi Y. (2024). Effects of Additional Oral Fosravuconazole l-lysine Ethanolate Therapy Following Inadequate Response to Initial Treatment for Onychomycosis: A Multicenter, Randomized Controlled Trial. J. Dermatol..

[33] Tsunemi Y., Naka W. (2025). Exploratory Study on Short-term Administration of Oral Fosravuconazole for Tinea Pedis. J. Dermatol..

[34] Miyata A., Kimura U., Noguchi H., Hiruma M., Kano R., Suga Y. (2025). Tinea Capitis Caused by *Trichophyton tonsurans* That Responded to Fosravuconazole. Med. Mycol. J..

[35] Saraswat A., Dogra S., Shenoy M., Verma S., K S., Ghate S., Ganjoo A., Aurangabadkar S., Tiwari A., Poojary S. (2024). Clinical Use of Super-Bioavailable Itraconazole for the Management of Dermatophytosis: Consensus Statement by Dermatologists from India via the Modified Delphi Technique. Dermatology.

[36] Dhoot D., Jain G.K., Manjhi M., Kesharwani P., Mahadkar N., Barkate H. (2023). Pharmacokinetic and Clinical Comparison of Super-Bioavailable Itraconazole and Conventional Itraconazole at Different Dosing in Dermatophytosis. Drugs Context.

[37] Das S., Chakraborty P., Kumar I., Roy S., Ghosh B., Mete R., Agarwal S., Sharma M., Bose A., Mondal S. (2025). Comparative Clinical Superiority of SUBA Itraconazole with Safety Evaluation in the Light of Pharmacokinetic and Pharmacodynamics Investigation in Healthy Indian Human Volunteers. Toxicol. Anal. Clin..

[38] Shenoy M., De A., Shah B., Das A., Saraswat A., Lahiri K., Yadav S., Sarda A., Chakraborty D., J D. (2023). Efficacy of Super-Bioavailable Itraconazole and Conventional Itraconazole at Different Dosing Regimens in Glabrous Tinea Infection—A Randomized Clinical Trial. Drug Des. Devel. Ther..

[39] Patel N.H., Sardana K., Shenoy M.M., Rengasamy M., Khurana A., Ghate S., Venkata C.K., Marfatiya Y., Bhunia D., Jayaraman J. (2024). IADVL SIG Recalcitrant Dermatophytosis Position Statement on Super Bioavailable Itraconazole. Indian Dermatol. Online J..

[40] Shah B., Mistry D., Jairam D., Kansara K., Pandya R., Vasani P., Dhoot D., Mahadkar N., Bhushan S., Barkate H. (2023). Comparative Efficacy of Super Bioavailable Itraconazole Capsules 50 mg vs 65 mg Twice Daily in the Management of Glabrous Tinea. Infect. Drug Resist..

[41] Mohapatra L., Dixit N., Agrawal I., Kar B.R., Singh B.S.T.P. (2023). Comparison of Efficacy and Safety between Super-Bioavailable Itraconazole and Conventional Itraconazole in the Treatment of Tinea Infection of Glabrous Skin—A Randomised Observer-Blinded Pilot Study. J. Pure Appl. Microbiol..

[42] Shenoy M., De A., Shah B., Das A., Saraswat A., Lahiri K., Dhoot D. (2023). Comparative Efficacy and Safety of Super-Bioavailable Itraconazole-130 mg Once Daily in Obese and Non-Obese Patients of Glabrous Tinea. Indian Dermatol. Online J..

[43] Shenoy M., Dhoot D., Mahajan H., Barkate H. (2021). An Open-Label, Randomized, Double-Arm Clinical Trial to Compare the Effectiveness and Safety of Super Bioavailable Itraconazole Capsules and Itraconazole Capsules in the Management of Dermatophytosis in India. Clin. Cosmet. Investig. Dermatol..

[44] Dhabal A., Das N.K., Sil A. (2023). Symmetric Drug-Related Intertriginous and Flexural Exanthema Due to Super-Bioavailable Itraconazole. Indian J. Pharmacol..

[45] Tyagi H., Goel S., Puri A., Singla I., Rathore A. (2025). Comparative Efficacy of Three Oral Itraconazole Formulations in Superficial Dermatophytosis. Mycoses.

[46] Tsang C.-C., Hui T.W.S., Lee K.-C., Chen J.H.K., Ngan A.H.Y., Tam E.W.T., Chan J.F.W., Wu A.L., Cheung M., Tse B.P.H. (2016). Genetic Diversity of *Aspergillus* Species Isolated from Onychomycosis and *Aspergillus hongkongensis* Sp. Nov., with Implications to Antifungal Susceptibility Testing. Diagn. Microbiol. Infect. Dis..

[47] Gupta C., Jongman M., Das S., Snehaa K., Bhattacharya S.N., Seyedmousavi S., van Diepeningen A.D. (2016). Genotyping and In Vitro Antifungal Susceptibility Testing of *Fusarium* Isolates from Onychomycosis in India. Mycopathologia.

[48] Singh S.K., Patwa D.K., Tilak R., Das A., Singh T.B. (2019). In Vitro Susceptibility of Dermatophytes to Oral Antifungal Drugs and Amphotericin B in Uttar Pradesh, India. Indian J. Dermatol. Venereol. Leprol..

[49] Ansari S., Ahmadi B., Norouzi M., Ansari Z., Afsarian M.H., Lotfali E., Rezaei-Matehkolaei A. (2019). *Epidermophyton floccosum*: Nucleotide Sequence Analysis and Antifungal Susceptibility Testing of 40 Clinical Isolates. J. Med. Microbiol..

[50] Salehi Z., Fatahi N., Taran M., Izadi A., Badali H., Hashemi S.J., Rezaie S., Daie Ghazvini R., Ghaffari M., Aala F. (2020). Comparison of in Vitro Antifungal Activity of Novel Triazoles with Available Antifungal Agents against Dermatophyte Species Caused Tinea Pedis. J. Mycol. Med..

[51] Shaw D., Singh S., Dogra S., Jayaraman J., Bhat R., Panda S., Chakrabarti A., Anjum N., Chowdappa A., Nagamoti M. (2020). MIC and Upper Limit of Wild-Type Distribution for 13 Antifungal Agents against a *Trichophyton mentagrophytes*-*Trichophyton interdigitale* Complex of Indian Origin. Antimicrob. Agents Chemother..

[52] Ebert A., Monod M., Salamin K., Burmester A., Uhrlaß S., Wiegand C., Hipler U.-C., Krüger C., Koch D., Wittig F. (2020). Alarming India-Wide Phenomenon of Antifungal Resistance in Dermatophytes: A Multicentre Study. Mycoses.

[53] Nenoff P., Verma S.B., Ebert A., Süß A., Fischer E., Auerswald E., Dessoi S., Hofmann W., Schmidt S., Neubert K. (2020). Spread of Terbinafine-Resistant *Trichophyton mentagrophytes* Type VIII (India) in Germany—“The Tip of the Iceberg?”. J. Fungi.

[54] Łagowski D., Gnat S., Nowakiewicz A., Osińska M., Dyląg M. (2020). Intrinsic Resistance to Terbinafine among Human and Animal Isolates of *Trichophyton mentagrophytes* Related to Amino Acid Substitution in the Squalene Epoxidase. Infection.

[55] Xu X., Naseri A., Houbraken J., Akbari F., Wang X., Zhao R., Zhang H., Najafzadeh M.J., Deng S. (2021). Identification and in Vitro Antifungal Susceptibility of Causative Agents of Onychomycosis Due to *Aspergillus* Species in Mashhad, Iran. Sci. Rep..

[56] Ansari S., Ahmadi B., Hedayati M.T., Nouripour-Sisakht S., Taghizadeh-Armaki M., Fathi M., Deravi N., Shokoohi G.-R., Rezaei-Matehkolaei A. (2021). Investigation of in Vitro Antifungal Susceptibility Testing and Genetic Diversity of Clinical Isolates of *Trichophyton benhamiae* and *Trichophyton eriotrephon* in Iran. Mycoses.

[57] Aneke C.I., Rhimi W., Hubka V., Otranto D., Cafarchia C. (2021). Virulence and Antifungal Susceptibility of *Microsporum canis* Strains from Animals and Humans. Antibiotics.

[58] Mahajan S., Tilak R., Kaushal S., Mishra R., Pandey S. (2017). Clinico-Mycological Study of Dermatophytic Infections and Their Sensitivity to Antifungal Drugs in a Tertiary Care Center. Indian J. Dermatol. Venereol. Leprol..

[59] Chang W., Bao F., Wang Z., Liu H., Zhang F. (2021). Comparison of the Sensititre YeastOne^®^ and CLSI M38-A2 Microdilution Methods in Determining the Activity of Nine Antifungal Agents against Dermatophytes. Mycoses.

[60] Ngo T.M.C., Ton Nu P.A., Le C.C., Vo M.T., Ha T.N.T., Do T.B.T., Nguyen P.V., Tran Thi G., Santona A. (2022). Nannizzia Incurvata in Hue City—Viet Nam: Molecular Identification and Antifungal Susceptibility Testing. J. Mycol. Med..

[61] Pashootan N., Shams-Ghahfarokhi M., Chaichi Nusrati A., Salehi Z., Asmar M., Razzaghi-Abyaneh M. (2022). Phylogeny, Antifungal Susceptibility, and Point Mutations of SQLE Gene in Major Pathogenic Dermatophytes Isolated from Clinical Dermatophytosis. Front. Cell. Infect. Microbiol..

[62] Sharma S., Maheshwari M., Thakur R., Sah S.P., Chauhan S. (2022). Antifungal Susceptibility Testing of Five Antifungal Agents against Clinically Isolated Dermatophytes Species from a Tertiary Care Hospital in Northern India. J. Clin. Diagn. Res..

[63] Cañete-Gibas C.F., Mele J., Patterson H.P., Sanders C.J., Ferrer D., Garcia V., Fan H., David M., Wiederhold N.P. (2023). Terbinafine-Resistant Dermatophytes and the Presence of *Trichophyton indotineae* in North America. J. Clin. Microbiol..

[64] Kashif S., Khan Khoso B., Zeeshan F., Khalid M., Hussain W., Uddin F. (2023). Antifungal Susceptibility Pattern of the Candida Species in Nail Infections. Med. Forum Mon..

[65] Zhang R., Song Z., Su X., Li T., Xu J., He X., Yang Y., Chang B., Kang Y. (2024). Molecular Epidemiology and Antifungal Susceptibility of Dermatophytes and Candida Isolates in Superficial Fungal Infections at a Grade A Tertiary Hospital in Northern China. Med. Mycol..

[66] Ngo T.M.C., Santona A., Ton Nu P.A., Cao L.C., Tran Thi G., Do T.B.T., Ha T.N.T., Vo Minh T., Nguyen P.V., Ton That D.D. (2024). Detection of Terbinafine-Resistant *Trichophyton indotineae* Isolates within the *Trichophyton mentagrophytes* Species Complex Isolated from Patients in Hue City, Vietnam: A Comprehensive Analysis. Med. Mycol..

[67] Xie W., Kong X., Zheng H., Mei H., Ge N., Liu W., Liang G., Li X. (2025). In Vitro Susceptibility Profiles of 16 Antifungal Drugs against *Trichophyton indotineae*. Microbiol. Spectr..

[68] Shao Y., Shao J., de Hoog S., Verweij P., Bai L., Richardson R., Richardson M., Wan Z., Li R., Yu J. (2025). Emerging Antifungal Resistance in *Trichophyton mentagrophytes*: Insights from Susceptibility Profiling and Genetic Mutation Analysis. Emerg. Microbes Infect..

[69] Rosa P.D., Heidrich D., Corrêa C., Scroferneker M.L., Vettorato G., Fuentefria A.M., Goldani L.Z. (2017). Genetic Diversity and Antifungal Susceptibility of *Fusarium* Isolates in Onychomycosis. Mycoses.

[70] McTaggart L.R., Cronin K., Ruscica S., Patel S.N., Kus J.V. (2025). Emergence of Terbinafine-Resistant *Trichophyton indotineae* in Ontario, Canada, 2014–2023. J. Clin. Microbiol..

[71] Ngo T.M.C., Ton That D.D., Ton Nu P.A., Chi Cao L., Tran Thi G., Do T.B.T., Ha T.N.T., Tiep Vo M., Nguyen P.V., Mai B.H.A. (2025). A Notable Azole-Nonsusceptible *Candida orthopsilosis* in the *Candida parapsilosis* Complex Isolated from Onychomycosis in Hue City, Central Vietnam. Med. Mycol..

[72] Applebach E., Amburgey-Crovetti K., Bakotic W.L., Adhikari T.B., Wiederhold N.P., Hodges S., Steen T.Y., Calderone R., Li D. (2025). Prevalence and Diversity of Antifungal Resistance in *Fusarium* Isolates across Clinical and Agricultural Settings in the United States. Antimicrob. Agents Chemother..

[73] Tang C., Kong X., Jansen J., Vossgroene K., Vu T.-L.-A., Oberheitmann B., Tehupeiory-Kooreman M., Zhou S., Zhou X., Tsui C.K.-M. (2025). Utility of MALDI-ToF MS for Recognition and Antifungal Susceptibility of Nannizzia, an Underestimated Group of Dermatophytes. Mycoses.

[74] Das S., Rawat D., Kaur R., Mendiratta V., Singh P.K. (2025). Comparative Analysis of In Vitro Susceptibility Profile of Dermatophytes Against 8 Antifungal Agents: A Cross-Sectional Study. Indian J. Dermatol..

[75] Kong X., Xie W., Fu M., Feng P., Li Z., Liu H., Tong Z., Abliz P., Jiang Y., Yang L. (2026). Antifungal Resistance of the *Trichophyton mentagrophytes*/*Trichophyton interdigitale* Species Complex: Insights from the China Antifungal Resistance Dermatophytes Surveillance Network Study (CARDS). J. Eur. Acad. Dermatol. Venereol..

[76] Shahid M., Raheem T., Zaka S., Farooqi J., Naqvi S.F., Talat H., Ghanchi N., Zafar A., Jabeen K. (2026). SQLE Mutations and Antifungal Susceptibility Profile of *Trichophyton* Species Isolated from Patients with Recalcitrant Dermatophytosis: A Laboratory-Based Study from Pakistan. Med. Mycol..

[77] Nofal A., Fawzy M.M., El-Hawary E.E. (2020). Successful Treatment of Resistant Onychomycosis with Voriconazole in a Liver Transplant Patient. Dermatol. Ther..

[78] Firooz A., Lotfali E., Fattahi M., Fattahi M., Miramin Mohammadi A., Shahrzad Kavkani M. (2021). A Case of Terbinafine-Resistant Tinea Cruris Caused by *Trichophyton tonsurans*. Case Rep. Dermatol. Med..

[79] Khattab F., Elkholy B.M., Taha M., Abd-Elbaset A., Fawzy M. (2022). Voriconazole Is Superior to Combined Itraconazole/Isotretinoin Therapy and Itraconazole Monotherapy in Recalcitrant Dermatophytosis. Mycoses.

[80] Muangkaew W., Wongsuk T., Luplertlop N. (2017). Common Dermatophytes and in Vitro Anti-Fungal Susceptibility Testing in Patients Attending the Dermatological Clinic at the Hospital for Tropical Medicine, Bangkok. New Microbiol..

[81] Khurana A., Agarwal A., Agrawal D., Sardana K., Singh A., Chowdhary A. (2022). Multidrug Resistant Tinea Corporis/Cruris: Response to Voriconazole. J. Mycol. Med..

[82] Chandrashekar B., Poojitha D. (2022). Evaluation of Efficacy and Safety of Oral Voriconazole in the Management of Recalcitrant and Recurrent Dermatophytosis. Clin. Exp. Dermatol..

[83] Shahzad M.K., Hassan T., Tahir R., Jawaid K., Khan M.F., Naveed M.A. (2022). Efficacy of Oral Voriconazole in the Treatment of Dermatophyte Infections (Tinea Corporis and Cruris). Pak. J. Med. Health Sci..

[84] Dashti Y., Alobaid K., Al-Rashidi S., Dashti M., AbdulMoneim M.H., Al-Enezi M., Abou-Chakra N., Jørgensen K.M. (2023). Autochthonous Case of *Trichophyton indotineae* in Kuwait. J. Mycol. Med..

[85] Ali M., Ahmed N., Khan S., kiran A. (2024). Efficacy of Oral Voriconazole versus Oral Itraconazole, in the Treatment of Dermatophyte Infections. J. Ayub Med. Coll. Abbottabad.

[86] Berstecher N., Burmester A., Gregersen D.M., Tittelbach J., Wiegand C. (2024). *Trichophyton indotineae* Erg1Ala448Thr Strain Expressed Constitutively High Levels of Sterol 14-α Demethylase Erg11B MRNA, While Transporter MDR3 and Erg11A MRNA Expression Was Induced After Addition of Short Chain Azoles. J. Fungi.

[87] Gupta A.K., Talukder M., Cooper E.A., Magal L., Shemer A. (2025). Efficacy and Safety of Voriconazole for Difficult-to-Treat Distal Lateral Subungual Onychomycosis (DLSO). Pathogens.

[88] Tan X.T., Saifuddin S.A.S., Mohamed Yusof N.I.S., Teo H.G., Tang M.M. (2025). Recalcitrant Dermatophytosis Due to *Trichophyton indotineae*: A Case Series from Sarawak, Malaysia. Med. Mycol. Case Rep..

[89] Luong M.-L., Al-Dabbagh M., Groll A.H., Racil Z., Nannya Y., Mitsani D., Husain S. (2016). Utility of Voriconazole Therapeutic Drug Monitoring: A Meta-Analysis. J. Antimicrob. Chemother..

[90] Chai S., Zhan J.-L., Zhao L.-M., Liu X.-D. (2022). Safety of Triazole Antifungals: A Pharmacovigilance Study from 2004 to 2021 Based on FAERS. Ther. Adv. Drug Saf..

[91] Salehi Z., Shams-Ghahfarokhi M., Razzaghi-Abyaneh M. (2018). Antifungal Drug Susceptibility Profile of Clinically Important Dermatophytes and Determination of Point Mutations in Terbinafine-Resistant Isolates. Eur. J. Clin. Microbiol. Infect. Dis..

[92] Clary R.T., Deja E., Rittmann B., Bearman G. (2025). Impact of Voriconazole Therapeutic Drug Monitoring on Adverse Effects and Clinical Outcomes: A Literature Review. Curr. Infect. Dis. Rep..

[93] Akdogan N., Kaymak M.K., Bosnak C., Sonmezer M.C., Gulmez D., Polat C., Akdagli S.A. (2026). Clinical Characteristics, Genomic Sequencing, and Treatment Response of 13 Cases Infected with the Emerging Pathogen, *Trichopyhton indotineae*, from Türkiye. Int. J. Dermatol..

[94] Giovannini S., Weibel L., Schittek B., Sinnberg T., Schaller M., Lemberg C., Fehrenbacher B., Biesemeier A., Nordin R., Ivanova I. (2024). Skin Cancer Induction by the Antimycotic Drug Voriconazole Is Caused by Impaired DNA Damage Detection Due to Chromatin Compaction. J. Investig. Dermatol..

[95] Ike J.I., Smith I.T., Mosley D., Madden C., Grossarth S., Halle B.R., Lewis A., Mentch F., Hakonarson H., Bastarache L. (2024). Voriconazole Metabolism Is Associated with the Number of Skin Cancers per Patient. Arch. Dermatol. Res..

[96] Tang H., Shi W., Song Y., Han J. (2019). Voriconazole Exposure and Risk of Cutaneous Squamous Cell Carcinoma among Lung or Hematopoietic Cell Transplant Patients: A Systematic Review and Meta-Analysis. J. Am. Acad. Dermatol..

[97] Kolaitis N.A., Duffy E., Zhang A., Lo M., Barba D.T., Chen M., Soriano T., Hu J., Nabili V., Saggar R. (2017). Voriconazole Increases the Risk for Cutaneous Squamous Cell Carcinoma after Lung Transplantation. Transpl. Int..

[98] Mansh M., Binstock M., Williams K., Hafeez F., Kim J., Glidden D., Boettger R., Hays S., Kukreja J., Golden J. (2016). Voriconazole Exposure and Risk of Cutaneous Squamous Cell Carcinoma, *Aspergillus* Colonization, Invasive Aspergillosis and Death in Lung Transplant Recipients. Am. J. Transplant..

[99] Naseer M., Devi V., Tahira, Naz Khan K., Asad N., Azhar A., Iqbal Asif M., Maheshwary N., Ahmed A., Athar Khan M. (2025). Efficacy and Safety of Voriconazole in the Treatment of Tinea Corporis and Cruris Infections. J. Pak. Assoc. Dermatol..

[100] Nácher A., Peris J.-E., Díez-Sales O., Taléns-Visconti R., Manca M.L., Manconi M., Usach I. (2026). Next-Generation Topical Antifungal Therapy: Biocompatible Voriconazole Nanostructured Lipid Carriers with Enhanced Skin Penetration for Cutaneous Candidiasis. J. Drug Deliv. Sci. Technol..

[101] Yadav M., Raju B., Narendra G., Kaur J., Kumar M., Silakari O., Sapra B. (2025). Leveraging Machine Learning to Predict Drug Permeation: Impact of Menthol and Limonene as Enhancers. Mol. Divers..

[102] Tupaki-Sreepurna A., Thanneru V., Natarajan S., Sharma S., Gopi A., Sundaram M., Kindo A.J. (2018). Phylogenetic Diversity and In Vitro Susceptibility Profiles of Human Pathogenic Members of the *Fusarium* Fujikuroi Species Complex Isolated from South India. Mycopathologia.

[103] Gackowski M., Froelich A., Kordyl O., Długaszewska J., Kamińska D., Schneider R., Osmałek T. (2025). Formulation Studies on Microemulsion-Based Polymer Gels Loaded with Voriconazole for the Treatment of Skin Mycoses. Pharmaceutics.

[104] Bouchand C., Nguyen D., Secretan P.-H., Vidal F., Guery R., Auvity S., Cohen J.F., Lanternier F., Lortholary O., Cisternino S. (2020). Voriconazole Topical Cream Formulation: Evidence for Stability and Antifungal Activity. Int. J. Antimicrob. Agents.

[105] Shah M.K.A., Azad A.K., Nawaz A., Ullah S., Latif M.S., Rahman H., Alsharif K.F., Alzahrani K.J., El-Kott A.F., Albrakati A. (2021). Formulation Development, Characterization and Antifungal Evaluation of Chitosan NPs for Topical Delivery of Voriconazole In Vitro and Ex Vivo. Polymers.

[106] Shen T., Li M., Tian B., Liu W., Chu L., Yu P., Zhou H., Han Y., Ding C., Sai S. (2024). Calcofluor White-Phosphatidylethanolamine Conjugate-Enhanced Ethosomal Delivery of Voriconazole for Targeting Candida Albicans. Int. J. Nanomed..

[107] Faisal W., Soliman G.M., Hamdan A.M. (2018). Enhanced Skin Deposition and Delivery of Voriconazole Using Ethosomal Preparations. J. Liposome Res..

[108] Nácher A., Peris J.-E., Taléns-Visconti R., Díez-Sales O., Manca M.L., Manconi M., Usach I. (2025). Novel Voriconazole-Loaded Hyalurosomes Optimized for Enhanced Skin Penetration and Antifungal Activity against Candida Albicans. Drug Deliv. Transl. Res..

[109] Amareshwar S., Abbaraju Krishna A. (2024). Development and Evaluation of Voriconazole Loaded Invasomes Gel for Enhanced Antifungal Activity. Int. J. Pharm. Qual. Assur..

[110] Farooq M., Usman F., Zaib S., Shah H.S., Jamil Q.A., Akbar Sheikh F., Khan A., Rabea S., Hagras S.A.A., El-Saber Batiha G. (2022). Fabrication and Evaluation of Voriconazole Loaded Transethosomal Gel for Enhanced Antifungal and Antileishmanial Activity. Molecules.

[111] Santos G.A., Angelo T., Andrade L.M., Silva S.M.M., Magalhães P.O., Cunha-Filho M., Gelfuso G.M., Taveira S.F., Gratieri T. (2018). The Role of Formulation and Follicular Pathway in Voriconazole Cutaneous Delivery from Liposomes and Nanostructured Lipid Carriers. Colloids Surf. B Biointerfaces.

[112] Krawczyk-Santos A.P., da Rocha P.B.R., Kloppel L.L., Souza B.D.S., Anjos J.L.V., Alonso A., de Faria D.L.A., Gil O.M., Gratieri T., Marreto R.N. (2021). Enhanced Nail Delivery of Voriconazole-Loaded Nanomicelles by Thioglycolic Acid Pretreatment: A Study of Protein Dynamics and Disulfide Bond Rupture. Int. J. Pharm..

[113] Sav H., Baris A., Turan D., Altinbas R., Sen S. (2018). The Frequency, Antifungal Susceptibility and Enzymatic Profiles of Candida Species in Cases of Onychomycosis Infection. Microb. Pathog..

[114] Gueneau R., Joannard B., Haddad N., Alby F., Jullien V., Schlatter J., Cotteret C., Bougnoux M.E., Lanternier F., Laroche L. (2022). Extensive Dermatophytosis Caused by Terbinafine-Resistant *Trichophyton indotineae*, Successfully Treated with Topical Voriconazole. Int. J. Antimicrob. Agents.

[115] Sardana K., Kaur R., Arora P., Goyal R., Ghunawat S. (2018). Is Antifungal Resistance a Cause for Treatment Failure in Dermatophytosis: A Study Focused on Tinea Corporis and Cruris from a Tertiary Centre?. Indian Dermatol. Online J..

[116] Rudramurthy S.M., Shankarnarayan S.A., Dogra S., Shaw D., Mushtaq K., Paul R.A., Narang T., Chakrabarti A. (2018). Mutation in the Squalene Epoxidase Gene of *Trichophyton interdigitale* and *Trichophyton rubrum* Associated with Allylamine Resistance. Antimicrob. Agents Chemother..

[117] Warrilow A.G.S., Parker J.E., Price C.L., Garvey E.P., Hoekstra W.J., Schotzinger R.J., Wiederhold N.P., Nes W.D., Kelly D.E., Kelly S.L. (2017). The Tetrazole VT-1161 Is a Potent Inhibitor of *Trichophyton rubrum* through Its Inhibition of T. Rubrum CYP51. Antimicrob. Agents Chemother..

[118] Elewski B., Brand S., Degenhardt T., Curelop S., Pollak R., Schotzinger R., Tavakkol A., Alonso-Llamazares J., Ashton S.J., Bhatia N. (2021). A Phase II, Randomized, Double-blind, Placebo-controlled, Dose-ranging Study to Evaluate the Efficacy and Safety of VT-1161 Oral Tablets in the Treatment of Patients with Distal and Lateral Subungual Onychomycosis of the Toenail. Br. J. Dermatol..

[119] Takei-Masuda N., Iida M., Ohyama M., Kaneda K., Ueda K., Tabata Y. (2025). Structure-Activity Relationship Studies of ME1111, a Novel Antifungal Agent for Topical Treatment of Onychomycosis. J. Antibiot..

[120] Tabata Y., Takei-Masuda N., Kubota N., Takahata S., Ohyama M., Kaneda K., Iida M., Maebashi K. (2016). Characterization of Antifungal Activity and Nail Penetration of ME1111, a New Antifungal Agent for Topical Treatment of Onychomycosis. Antimicrob. Agents Chemother..

[121] Takahata S., Kubota N., Takei-Masuda N., Yamada T., Maeda M., Alshahni M.M., Abe S., Tabata Y., Maebashi K. (2016). Mechanism of Action of ME1111, a Novel Antifungal Agent for Topical Treatment of Onychomycosis. Antimicrob. Agents Chemother..

[122] Hui X., Jung E.C., Zhu H., Maibach H.I. (2017). Antifungal ME1111 In Vitro Human Onychopharmacokinetics. Drug Dev. Ind. Pharm..

[123] Kubota-Ishida N., Takei-Masuda N., Kaneda K., Nagira Y., Chikada T., Nomoto M., Tabata Y., Takahata S., Maebashi K., Hui X. (2018). In Vitro Human Onychopharmacokinetic and Pharmacodynamic Analyses of ME1111, a New Topical Agent for Onychomycosis. Antimicrob. Agents Chemother..

[124] Ghannoum M., Chaturvedi V., Diekema D., Ostrosky-Zeichner L., Rennie R., Walsh T., Wengenack N., Fothergill A., Wiederhold N. (2016). Multilaboratory Evaluation of In Vitro Antifungal Susceptibility Testing of Dermatophytes for ME1111. J. Clin. Microbiol..

[125] Long L., Hager C., Ghannoum M. (2016). Evaluation of the Efficacy of ME1111 in the Topical Treatment of Dermatophytosis in a Guinea Pig Model. Antimicrob. Agents Chemother..

[126] Takei-Masuda N., Nagira Y., Kubota-Ishida N., Chikada T., Tabata Y., Maebashi K. (2024). Antidermatophyte Activity and PK/PD of ME1111 in a Guinea Pig Model of Tinea Corporis. J. Antibiot..

[127] Mirbzadeh Ardakani E., Sharifirad A., Pashootan N., Nayebhashemi M., Zahmatkesh M., Enayati S., Razzaghi-Abyaneh M., Khalaj V. (2021). Olorofim Effectively Eradicates Dermatophytes In Vitro and In Vivo. Antimicrob. Agents Chemother..

[128] Singh A., Singh P., Meis J.F., Chowdhary A. (2021). In Vitro Activity of the Novel Antifungal Olorofim against Dermatophytes and Opportunistic Moulds Including *Penicillium* and *Talaromyces* Species. J. Antimicrob. Chemother..

[129] Liang T., Chen X., de Hoog G.S., Li L., Wang L., Wan Z., Yu J., Li R., Song Y. (2025). Antifungal Resistance Patterns of *Microsporum canis*: A 27-Year MIC Study in Mainland China. Mycoses.

[130] Perez A.P., Altube M.J., Schilrreff P., Apezteguia G., Celes F.S., Zacchino S., de Oliveira C.I., Romero E.L., Morilla M.J. (2016). Topical Amphotericin B in Ultradeformable Liposomes: Formulation, Skin Penetration Study, Antifungal and Antileishmanial Activity in Vitro. Colloids Surf. B Biointerfaces.

[131] Ahmad Nasrollahi S., Fattahi A., Naeimifar A., Lotfali E., Firooz A., Khamesipoor A., Skandari S.E., Miramin Mohammadi A. (2022). The in Vitro Effect of Nanoliposomal Amphotericin B against Two Clinically Important Dermatophytes. Int. J. Dermatol..

[132] Nosratabadi M., Akhtari J., Jaafari M.R., Yahyazadeh Z., Shokohi T., Haghani I., Farmani P., Barough R.E., Badali H., Abastabar M. (2024). In Vitro Activity of Nanoliposomal Amphotericin B against Terbinafine-Resistant *Trichophyton indotineae* Isolates. Int. Microbiol..

[133] Souza A.M.S., Ribeiro R.C.A., Pinheiro G.K.L.O., Pinheiro F.I., Oliveira W.N., Souza L.B.F.C., Silva A.L., Amaral-Machado L., Alencar É.N., Chaves G.M. (2021). Polishing the Therapy of Onychomycosis Induced by *Candida* spp.: Amphotericin B–Loaded Nail Lacquer. Pharmaceutics.

[134] Firooz A., Zamani S., Ghadrei A., Ayatollahi A., Tamimi P., Khamesipour A., Jafari M., Fattahi M. (2023). Evaluation of Efficacy and Safety of Topical Nanoliposomal Amphotericin B 0.4% Gel as a Potential Treatment for Onychomycosis: An Interventional Pilot Clinical Study. Dermatol. Ther..

[135] Peng K., Ammar A.A., Himawan A., Dai X., Duncan R., Gilmore B.F., Donnelly R.F., Vora L.K. (2025). Amphotericin B PLGA Nanoparticles Loaded Dissolving Microneedle Patches in Treating Cutaneous Fungal Infections. J. Drug Deliv. Sci. Technol..

[136] Zhou Y.B., Xiao Y.Y., Chao J.J., Ma L. (2021). In Vitro Activity of Allicin Alone and in Combination with Antifungal Drugs Against *Microsporum canis* Isolated from Patients with Tinea Capitis. Front. Med..

[137] Ke J., Yan Y. (2024). Allicin Attenuates UVB-Induced Photodamage of Keratinocytes by Inhibiting NLRP3 Inflammasomes and Activating the PI3K/Akt Pathway. Arch. Dermatol. Res..

[138] Pârvu M., Moţ C.A., Pârvu A.E., Mircea C., Stoeber L., Roşca-Casian O., Ţigu A.B. (2019). Allium Sativum Extract Chemical Composition, Antioxidant Activity and Antifungal Effect against *Meyerozyma guilliermondii* and *Rhodotorula mucilaginosa* Causing Onychomycosis. Molecules.

[139] Kherde G.S., Khaire R.D., Kunde V.D. (2025). Development and Optimization of Mesoporous Silica Nanoparticle Loaded Hydrogel of Allicin for Wound Healing. Int. J. Appl. Pharm..

[140] Mercer D.K., Robertson J.C., Miller L., Stewart C.S., O’Neil D.A. (2020). NP213 (Novexatin^®^): A Unique Therapy Candidate for Onychomycosis with a Differentiated Safety and Efficacy Profile. Med. Mycol..

[141] Mercer D.K., Stewart C.S., Miller L., Robertson J., Duncan V.M.S., O’Neil D.A. (2019). Improved Methods for Assessing Therapeutic Potential of Antifungal Agents against Dermatophytes and Their Application in the Development of NP213, a Novel Onychomycosis Therapy Candidate. Antimicrob. Agents Chemother..

[142] Haghani I., Ebrahimi F.K., Abastabar M., Barough R.E., Amiri F.T., Hedayati M.T., Vaseghi N., Javidnia J., Nosratabadi M., Yahyazadeh Z. (2025). A Novel Niosomal Gel for Topical Delivery of Miltefosine Against *Trichophyton indotineae* Dermatophytosis in Animal Model. Mycoses.

[143] Dastan N., Maghsood A.H., Hatam G., Jafari M., Motavallihaghi S.M., Derakhshanfar A., Ardeshiri H., Tamaddon A.M. (2025). Experimental Evaluation of the Therapeutic Effect of Niosomal Miltefosine Delivered via Polyvinylpyrrolidone—Hyaluronic Acid Microneedle Patches against Leishmania Major. Int. J. Biol. Macromol..

[144] Kavian Z., Alavizadeh S.H., Golmohamadzadeh S., Badiee A., Khamesipour A., Jaafari M.R. (2019). Development of Topical Liposomes Containing Miltefosine for the Treatment of Leishmania Major Infection in Susceptible BALB/c Mice. Acta Trop..

[145] Batool S., Zahid F., Ud-Din F.-, Naz S.S., Dar M.J., Khan M.W., Zeb A., Khan G.M. (2021). Macrophage Targeting with the Novel Carbopol-Based Miltefosine-Loaded Transfersomal Gel for the Treatment of Cutaneous Leishmaniasis: In Vitro and in Vivo Analyses. Drug Dev. Ind. Pharm..

[146] Abo-Elyazeed H., Soliman R., Hassan H., El-Seedy F.R., Aboul-Ella H. (2023). Development, Preparation, and Evaluation of a Novel Non-Adjuvanted Polyvalent Dermatophytes Vaccine. Sci. Rep..

[147] Gaballah E.Y., Borg T.M., Mohamed E.A. (2022). Hydroxypropyl Chitosan Nail Lacquer of Ciclopirox-PLGA Nanocapsules for Augmented in Vitro Nail Plate Absorption and Onychomycosis Treatment. Drug Deliv..

[148] Al-Obaidi H., Petraityte I., Hibbard T., Majumder M., Kalgudi R., Zariwala M.G. (2022). Antifungal Nanosuspensions with Surfactants and Silver for the Treatment of Onychomycosis. Eur. J. Pharm. Biopharm..

[149] Prasong W., Matthapan L., Lertrujiwanit K., Supcharoenkul S., Ongsri P., Kiratiwongwan R., Leeyaphan C., Bunyaratavej S. (2022). In Vitro Antifungal Activity of Plain Socks and Zinc Oxide Nanoparticle-Coated Socks. J. Am. Podiatr. Med. Assoc..

[150] Wang F., Yang P., Choi J.-S., Antovski P., Zhu Y., Xu X., Kuo T.-H., Lin L.-E., Kim D.N.H., Huang P.-C. (2018). Cross-Linked Fluorescent Supramolecular Nanoparticles for Intradermal Controlled Release of Antifungal Drug-A Therapeutic Approach for Onychomycosis. ACS Nano.

[151] Mahtab A., Anwar M., Mallick N., Naz Z., Jain G.K., Ahmad F.J. (2016). Transungual Delivery of Ketoconazole Nanoemulgel for the Effective Management of Onychomycosis. AAPS PharmSciTech.

[152] Mordorski B., Costa-Orlandi C.B., Baltazar L.M., Carreño L.J., Landriscina A., Rosen J., Navati M., Mendes-Giannini M.J.S., Friedman J.M., Nosanchuk J.D. (2017). Topical Nitric Oxide Releasing Nanoparticles Are Effective in a Murine Model of Dermal *Trichophyton rubrum* Dermatophytosis. Nanomedicine.

[153] Viswanathan K., Vaiyamalai R., Bharathi Babu D., Mala Priyadharshini M.L., Raman M., Dhinakarraj G. (2018). Ketoconazole-Conjugated ZnO Nanoparticles Based Semi-Solid Formulation and Study Their Impacts on Skin Disease. IET Nanobiotechnol..

[154] Permana A.D., Paredes A.J., Volpe-Zanutto F., Anjani Q.K., Utomo E., Donnelly R.F. (2020). Dissolving Microneedle-Mediated Dermal Delivery of Itraconazole Nanocrystals for Improved Treatment of Cutaneous Candidiasis. Eur. J. Pharm. Biopharm..

[155] Sadozai S.K., Khan S.A., Baseer A., Ullah R., Zeb A., Schneider M. (2022). In Vitro, Ex Vivo, and In Vivo Evaluation of Nanoparticle-Based Topical Formulation Against Candida Albicans Infection. Front. Pharmacol..

[156] Kumar N., Goindi S. (2021). Development and Optimization of Itraconazole-Loaded Solid Lipid Nanoparticles for Topical Administration Using High Shear Homogenization Process by Design of Experiments: In Vitro, Ex Vivo and In Vivo Evaluation. AAPS PharmSciTech.

[157] AbdelSamie S.M., Kamel A.O., Sammour O.A., Ibrahim S.M. (2016). Terbinafine Hydrochloride Nanovesicular Gel: In Vitro Characterization, Ex Vivo Permeation and Clinical Investigation. Eur. J. Pharm. Sci..

[158] Abobakr F.E., Fayez S.M., Elwazzan V.S., Sakran W. (2021). Effect of Different Nail Penetration Enhancers in Solid Lipid Nanoparticles Containing Terbinafine Hydrochloride for Treatment of Onychomycosis. AAPS PharmSciTech.

[159] Al-Janabi A.A.H.S., Bashi A.M. (2019). Development of a New Synthetic Xerogel Nanoparticles of Silver and Zinc Oxide against Causative Agents of Dermatophytoses. J. Dermatolog. Treat..

[160] Costa-Orlandi C.B., Mordorski B., Baltazar L.M., Mendes-Giannini M.J.S., Friedman J.M., Nosanchuk J.D., Friedman A.J. (2018). Nitric Oxide Releasing Nanoparticles as a Strategy to Improve Current Onychomycosis Treatments. J. Drugs Dermatol..

[161] Parsay S., Saeedi M., Abastabar M., Hedayati M.T., Rahimnia S.M., Gholizadeh N., Kazeminejad A., Morteza-Semnani K., Gashti R.Z., Asare-Addo K. (2025). A Double-Blind Randomised Clinical Trial of Terbinafine-Nanostructured Lipid Carriers: Should We Anticipate This Strategy for Effective Topical Treatment of Onychomycosis?. Mycoses.

[162] Yun S.W., Lee J.G., Kim C.H., Kim K.S. (2025). Enhanced Efinaconazole Permeation and Activity Against *Trichophyton rubrum* and *Trichophyton mentagrophytes* with a Self-Nanoemulsifying Drug Delivery System. Pharmaceutics.

[163] Puri V., Froelich A., Shah P., Pringle S., Chen K., Michniak-Kohn B. (2022). Quality by Design Guided Development of Polymeric Nanospheres of Terbinafine Hydrochloride for Topical Treatment of Onychomycosis Using a Nano-Gel Formulation. Pharmaceutics.

[164] Ullah K.H., Rasheed F., Naz I., Ul Haq N., Fatima H., Kanwal N., Ur-Rehman T. (2022). Chitosan Nanoparticles Loaded Poloxamer 407 Gel for Transungual Delivery of Terbinafine HCl. Pharmaceutics.

[165] Kareem H.A., Samaka H.M., Abdulridha W.M. (2021). Evaluation of the Effect of the Gold Nanoparticles Prepared by Green chemistry on the treatment of Cutaneous Candidiasis. Curr. Med. Mycol..

[166] Saag M.S., Dismukes W.E. (1988). Azole Antifungal Agents: Emphasis on New Triazoles. Antimicrob. Agents Chemother..

[167] Therapeutic Goods Administration LOZANOC (Itraconazole) Capsules. https://www.ebs.tga.gov.au/ebs/picmi/picmirepository.nsf/pdf?OpenAgent=&id=CP-2014-PI-01749-1.

[168] Pharmaceuticals and Medical Devices Agency (PMDA) NAILIN Capsules 100 mg. https://www.info.pmda.go.jp/go/interview/1/300089_6290007M1022_1_008_1F.pdf.

[169] U.S. Food and Drug Administration Prescribing Information for TOLSURA (Itraconazole Capsules). https://www.accessdata.fda.gov/drugsatfda_docs/label/2024/208901s004lbl.pdf.

[170] U.S. Food and Drug Administration Prescribing Information for VEFEND (Voriconazole) Tablets, Oral Suspension, Intravenous Injection. https://www.accessdata.fda.gov/scripts/cder/daf/index.cfm?event=overview.process&ApplNo=021266.

[171] Health Canada Product Monograph for VEFEND Tablets, Injection, and Oral Suspension. https://pdf.hres.ca/dpd_pm/00080603.PDF.

[172] European Medicines Agency VFEND: EPAR—Product Information. https://www.ema.europa.eu/en/documents/product-information/vfend-epar-product-information_en.pdf.

[173] Medicines and Healthcare products Regulatory Agency Summary of Product Characteristics: Voriconazole 50 mg Film-Coated Tablets. https://products.mhra.gov.uk/.

[174] U.S. Food and Drug Administration Prescribing Information for VIVJOA (Oteseconazole) Capsules. https://www.accessdata.fda.gov/drugsatfda_docs/label/2024/215888s002lbl.pdf.

[175] Wiederhold N.P. (2022). Pharmacodynamics, Mechanisms of Action and Resistance, and Spectrum of Activity of New Antifungal Agents. J. Fungi.

[176] Abuhelwa A.Y., Foster D.J.R., Mudge S., Hayes D., Upton R.N. (2015). Population Pharmacokinetic Modeling of Itraconazole and Hydroxyitraconazole for Oral SUBA-Itraconazole and Sporanox Capsule Formulations in Healthy Subjects in Fed and Fasted States. Antimicrob. Agents Chemother..

[177] Teaford H.R., Abu Saleh O.M., Villarraga H.R., Enzler M.J., Rivera C.G. (2020). The Many Faces of Itraconazole Cardiac Toxicity. Mayo Clin. Proc. Innov. Qual. Outcomes.

[178] Saunte D.M., Simmel F., Frimodt-Moller N., Stolle L.B., Svejgaard E.L., Haedersdal M., Kloft C., Arendrup M.C. (2007). In Vivo Efficacy and Pharmacokinetics of Voriconazole in an Animal Model of Dermatophytosis. Antimicrob. Agents Chemother..

[179] Warrilow A.G.S., Hull C.M., Parker J.E., Garvey E.P., Hoekstra W.J., Moore W.R., Schotzinger R.J., Kelly D.E., Kelly S.L. (2014). The Clinical Candidate VT-1161 Is a Highly Potent Inhibitor of Candida Albicans CYP51 but Fails to Bind the Human Enzyme. Antimicrob. Agents Chemother..

[180] Oliver J.D., Sibley G.E.M., Beckmann N., Dobb K.S., Slater M.J., McEntee L., du Pré S., Livermore J., Bromley M.J., Wiederhold N.P. (2016). F901318 Represents a Novel Class of Antifungal Drug That Inhibits Dihydroorotate Dehydrogenase. Proc. Natl. Acad. Sci. USA.

[181] Jacobs S.E., Zagaliotis P., Walsh T.J. (2021). Novel Antifungal Agents in Clinical Trials. F1000Research.

[182] Yamada Y., Azuma K. (1977). Evaluation of the in Vitro Antifungal Activity of Allicin. Antimicrob. Agents Chemother..

[183] Firat Y.H., Simanski M., Rademacher F., Schröder L., Brasch J., Harder J. (2014). Infection of Keratinocytes with Trichophytum Rubrum Induces Epidermal Growth Factor-Dependent RNase 7 and Human Beta-Defensin-3 Expression. PLoS ONE.

[184] U.S. Food and Drug Administration Prescribing Information for IMPAVIDO (Miltefosine) Capsules. https://www.accessdata.fda.gov/drugsatfda_docs/label/2025/204684s007s008lbl.pdf.

[185] Nosratabadi M., Akhtari J., Faeli L., Haghani I., Aghili S.R., Shokohi T., Hedayati M.T., Zarrinfar H., Mohammadi R., Najafzadeh M.J. (2022). In Vitro Antifungal Susceptibility Profile of Miltefosine against a Collection of Azole and Echinocandins Resistant *Fusarium* Strains. J. Fungi.

[186] Vila T.V.M., Chaturvedi A.K., Rozental S., Lopez-Ribot J.L. (2015). In Vitro Activity of Miltefosine against Candida Albicans under Planktonic and Biofilm Growth Conditions and In Vivo Efficacy in a Murine Model of Oral Candidiasis. Antimicrob. Agents Chemother..

[187] Haghani I., Akhtari J., Yahyazadeh Z., Espahbodi A., Kermani F., Javidnia J., Hedayati M.T., Shokohi T., Badali H., Rezaei-Matehkolaei A. (2023). Potential Inhibitory Effect of Miltefosine against Terbinafine-Resistant *Trichophyton indotineae*. Pathogens.

[188] Waldman A., Segal R., Berdicevsky I., Gilhar A. (2010). CD4+ and CD8+ T Cells Mediated Direct Cytotoxic Effect against *Trichophyton rubrum* and *Trichophyton mentagrophytes*. Int. J. Dermatol..

[189] Hay R.J., Calderon R.A., Mackenzie C.D. (1988). Experimental Dermatophytosis in Mice: Correlation between Light and Electron Microscopic Changes in Primary, Secondary and Chronic Infections. Br. J. Exp. Pathol..

[190] Faway É., Poirier W., Yamada T., Ozawa K., Monod M., Mignon B., Poumay Y. (2025). SUB6 Subtilisin Is Involved During the Initial Adhesion of *Trichophyton benhamiae* and *T. Mentagrophytes* onto Reconstructed Human Epidermis. JID Innov..

[191] Méhul B., Gu Z., Jomard A., Laffet G., Feuilhade M., Monod M. (2016). Sub6 (Tri r 2), an Onychomycosis Marker Revealed by Proteomics Analysis of *Trichophyton rubrum* Secreted Proteins in Patient Nail Samples. J. Investig. Dermatol..

[192] Woodfolk J.A., Platts-Mills T.A. (2001). Diversity of the Human Allergen-Specific T Cell Repertoire Associated with Distinct Skin Test Reactions: Delayed-Type Hypersensitivity-Associated Major Epitopes Induce Th1- and Th2-Dominated Responses. J. Immunol..

[193] Bressani V.O., Santi T.N., Domingues-Ferreira M., Almeida A., Duarte A.J.S., Moraes-Vasconcelos D. (2013). Characterization of the Cellular Immunity in Patients Presenting Extensive Dermatophytoses Due to *Trichophyton rubrum*. Mycoses.

[194] Kumar P., Das S., Tigga R., Pandey R., Bhattacharya S.N., Taneja B. (2021). Whole Genome Sequences of Two *Trichophyton indotineae* Clinical Isolates from India Emerging as Threats during Therapeutic Treatment of Dermatophytosis. 3 Biotech.

[195] Brand W., Noorlander C.W., Giannakou C., De Jong W.H., Kooi M.W., Park M.V., Vandebriel R.J., Bosselaers I.E., Scholl J.H., Geertsma R.E. (2017). Nanomedicinal Products: A Survey on Specific Toxicity and Side Effects. Int. J. Nanomed..

[196] Zhang N., Xiong G., Liu Z. (2022). Toxicity of Metal-Based Nanoparticles: Challenges in the Nano Era. Front. Bioeng. Biotechnol..

[197] Portugal J., Bedia C., Amato F., Juárez-Facio A.T., Stamatiou R., Lazou A., Campiglio C.E., Elihn K., Piña B. (2024). Toxicity of Airborne Nanoparticles: Facts and Challenges. Environ. Int..

[198] Aneke C.I., Rhimi W., Otranto D., Cafarchia C. (2020). Synergistic Effects of Efflux Pump Modulators on the Azole Antifungal Susceptibility of *Microsporum canis*. Mycopathologia.

[199] Liu L., Zhang X., Kayastha S., Tan L., Zhang H., Tan J., Li L., Mao J., Sun Y. (2022). A Preliminary in Vitro and in Vivo Evaluation of the Effect and Action Mechanism of 17-AAG Combined with Azoles Against Azole-Resistant *Candida* spp. Front. Microbiol..

[200] European Committee on Antimicrobial Susceptibility Testing Overview of Antifungal ECOFFs and Clinical Breakpoints for Yeasts, Moulds and Dermatophytes Using the EUCAST E.Def 7.4, E.Def 9.4 and E.Def 11.0 Procedures. https://www.eucast.org/fungi-afst/clinical-breakpoints-and-interpretation/clinical-breakpoint-table/.

